# Mode of Antibacterial Action of Tomatidine C3-Diastereoisomers

**DOI:** 10.3390/molecules29020343

**Published:** 2024-01-10

**Authors:** Jean-Philippe Langlois, Audrey Larose, Eric Brouillette, Julien A. Delbrouck, Pierre-Luc Boudreault, François Malouin

**Affiliations:** 1 Département de Biologie, Faculté des Sciences, Université de Sherbrooke, Sherbrooke, QC J1K 2R1, Canada; jean-philippe.langlois@usherbrooke.ca (J.-P.L.); audrey.larose@usherbrooke.ca (A.L.); eric.brouillette@usherbrooke.ca (E.B.); 2 Département de Pharmacologie et Physiologie, Faculté de Médecine et des Sciences de la Santé, Université de Sherbrooke, Sherbrooke, QC J1H 5N4, Canada; julien.delbrouck@usherbrooke.ca

**Keywords:** *Staphylococcus aureus*, *Escherichia coli*, ATP synthase inhibitor, tomatidine, antibiotic mode of action, bactericidal activity, membrane potential, reactive oxygen species, membrane fluidity

## Abstract

Tomatidine (TO) is a natural narrow-spectrum antibiotic acting on the *Staphylococcus aureus* small colony variant (SCV) with a minimal inhibitory concentration (MIC) of 0.06 µg/mL while it shows no activity against prototypical strains (MIC > 128 µg/mL). To expand the spectrum of activity of TO, the 3β-hydroxyl group was substituted with an ethane-1,2-diamine, resulting in two diastereoisomers, **TM-02** (C3-β) and **TM-03** (C3-α). These molecules are equally potent against prototypical *S. aureus* and *E. coli* strains (MIC 8 and 32 µg/mL, respectively), whereas **TM-02** is more potent against SCV (MIC 0.5 µg/mL) and hyperpermeable *E. coli* strains (MIC 1 µg/mL). The differences in their modes of action were investigated. We used membrane vesicles to confirm the inhibition of the bacterial ATP synthase, the documented target of TO, and measured effects on bacterial cell membranes. Both molecules inhibited *E. coli* ATP synthase, with K_i_ values of 1.1 µM and 3.5 µM for **TM-02** and **TM-03**, respectively, and the bactericidal effect of **TM-02** was linked to ATP synthase inhibition. Furthermore, **TM-02** had no major effect on the membrane fluidity and gradually reduced membrane potential. In contrast, **TM-03** caused structural damages to membranes and completely disrupted the membrane potential (>90%). We were successful in broadening the spectrum of activity of TO. C3-β-diastereoisomers may have more specific antibacterial action than C3-α.

## 1. Introduction

The World Health Organization (WHO) listed carbapenem-resistant *Enterobacteriaceae* and methicillin-resistant *Staphylococcus aureus* (MRSA) among bacteria that represent urgent and significant public health threats and for which new antibiotics are needed to tackle antibiotic resistance [1]. These bacteria cause thousands of deaths each year [2,3]. The MRSA is also able to adopt an alternative phenotype to evade antibiotic treatment and the immune system. This phenotype, the small colony variant (SCV), is particularly tolerant to aminoglycosides and is often found in the lungs of chronically infected cystic fibrosis patients [4,5]. As a result, the discovery of new antibiotics and, more specifically, new antibiotic classes, is essential for tackling drug-resistant *E. coli*, *S. aureus*, and SCVs.

Tomatidine (TO) is a natural antibiotic isolated from the tomato. This antibiotic is effective against the persistent *S. aureus* SCV phenotype [6]. The bacterial ATP synthase subunit c, which is encoded by the gene *atpE*, is the cellular target of TO [7] and, thus, inhibits the generation of ATP. Consequently, TO also affects the membrane potential, which leads to the production of a large amount of reactive oxygen species (ROS) [8]. Although this compound’s antibacterial activity is quite selective for the SCV phenotype, it was demonstrated to have a good synergy with aminoglycosides like gentamicin and tobramycin against non-SCV MRSA [9].

**Figure 1:**
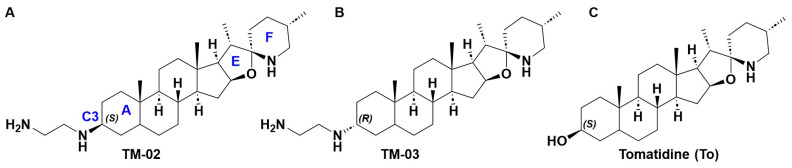
Structures of (**A**) **TM-02**, (**B**) **TM-03**, and (**C**) tomatidine. In (**A**), the nomenclature of the rings is indicated by a blue letter as for position C3.

The steroidal alkaloid scaffold of TO represents a novel type of molecule targeting bacterial ATP synthases, a clinically relevant underexploited target [10]. To expand the antibacterial spectrum of activity of TO, the C3-β-hydroxyl group was replaced with an ethane-1,2-diamine [10], yielding two diastereoisomers, **TM-02** (*aka* FcM) and **TM-03** (*aka* Fcm), with the diamine orientated out (C3-β) and into (C3-α) the plane, respectively (Figure 1) [7]. These molecules were also previously evaluated as a mix of **TM-02** and **TM-03**, known as FC04-100, which was shown to be effective against a variety of *Bacillales*, including *Listeria* and MRSA, and, like TO, to have a synergistic association with aminoglycosides [11]. Interestingly, this antibacterial activity was not limited to the SCV phenotype like it was for TO; FC04-100 also showed activity against prototypical strains [11]. Another important breakthrough for the C3-ethane-1,2-diamine derivatives of TO is that the spectrum of activity of these molecules now also extends to some Gram-negative bacteria such as *E. coli*, although this activity is relatively modest (minimal inhibitory concentration of 32 µg/mL) [7].

Published information on the two derivatives of TO revealed that the C3-β-ethane-1,2-diamine diastereoisomer (**TM-02**) was more active than **TM-03** against *S. aureus* SCVs. However, both **TM-02** and **TM-03** were equally effective against non-SCV *S. aureus* and *E. coli* [7]. In the hope of a future development of this class of antibiotics, more information is needed on the mode of action of these diastereoisomers. We hypothesized that the diamine’s spatial orientation could influence the specificity of the molecules for the ATP synthase target and/or could take part in a secondary mode of action involved in the activity against non-SCV bacteria. In this study, we compared and contrasted the primary and possibly secondary modes of action of the two C3-diastereoisomers **TM-02** and **TM-03**. The primary effect of these compounds on the bacterial ATP synthase was studied using purified bacterial membrane vesicles. Moreover, in order to explore a possible secondary mechanism of action, assays were carried out to establish the influence of these molecules on membrane fluidity, the bacterial membrane potential, and ROS production. We provide evidence that the bactericidal action of **TM-02** is linked to its high affinity for the ATP synthase and that **TM-03** compensates for its lower affinity for the primary target by a stronger interaction with bacterial membranes.

## 2. Results

### 2.1. Antibiotic Susceptibility

The antibiotic susceptibility profiles of all strains used in this study are reported in Table 1. As expected, the two *S. aureus* SCV strains (both Δ*hemB*) known to be hypersusceptible to ATP synthase inhibitors such as TO [8] were also more susceptible to both analog diastereoisomers (C3-β and C3-α) as compared to their parental strain (ATCC 29213 or Newbould). For *E. coli*, the hyperpermeable mutant MC4100-alt*lptD* is a strain that is generally more susceptible to antibiotics and is even susceptible to vancomycin, a large antibiotic which normally lacks activity against Gram-negative bacteria due to the outer membrane permeability barrier. This strain was also more susceptible to both diastereoisomers, indicating that there may be a permeability component limiting the access of the TM-compounds to the cellular target (i.e., ATP synthase) in the *E. coli* parental strain (MC4100). Interestingly, hypersusceptible strains (for example, *S. aureus* 29213Δ*hemB* and *E. coli* MC4100-alt*lptD*) were inhibited at lower concentrations by **TM-02** compared to **TM-03** (0.5 and 4 vs. 1 and 8 µg/mL, respectively, for **TM-02** vs. **TM-03**) while their parental strains were affected equally by both diastereoisomers (8 and 32 µg/mL, respectively, for *S. aureus* 29213 and *E. coli* MC4100 strains). For *B. subtilis* str. 168, a large Gram-positive bacterium which was used for microscopic imaging purposes, we also measured an MIC of 8 µg/mL.

When the expected cellular target of the TO analogs, the ATP synthase subunit c (*atpE*), was overexpressed in either *S. aureus* NewbouldΔ*hemB* or *E. coli* MC4100-alt*lptD*, we observed an increase in MIC for both **TM-02** and **TM-03** (Table 1). For *S. aureus* NewbouldΔ*hemB* overexpressing *atpE* (strain containing pCN36-*atpE*), we observed an increase in MIC for **TM-02** from 0.06 to 8 µg/mL and from 2 to 64 µg/mL for **TM-03**. Similarly, the MIC for the *E. coli* MC4100-alt*lptD* strain overexpressing *atpE* (strain containing pAC027-ColE1-*atpE*) increased from 1 and 8 to 64μg/mL for **TM-02** and **TM-03**, respectively.

The MIC of **TM-02** and **TM-03** for a TO-resistant *S. aureus* SCV strain (i.e., NewbouldΔ*hemB*_*atpE*G149V) having an SNP in *atpE* [7] increased from 0.06 to 2 µg/mL and from 2 to 8 µg/mL, respectively (Table 1). Thus, an SNP in *atpE* does not confer full resistance to either **TM-02** or **TM-03** but significantly reduces the hypersusceptibility of the SCV strain.

Hence, results from antibiotic susceptibility tests suggest that the ATP synthase is the main target for both **TM-02** and **TM-03**, as they are more effective against strains hypersusceptible to ATP synthase inhibitors and because the overexpression of the target (*atpE*) or a mutation in *atpE* reduced their inhibitory activity. On the other hand, results possibly indicate a secondary mode of action that is independent of their principal cellular target as the TO analogs still show some inhibitory activity against the *atpE* (G149V) and overexpression mutants, which is not seen in the case of TO [7].

### 2.2. ***TM-02*** Is More Potent at Inhibiting the ATP Synthase

As both diastereomers target the bacterial ATP synthase, we sought to determine if there was a notable difference in their potency by comparing their inhibition constants (K_i_) (Table 2). Using *E. coli* membrane vesicles as a source of ATP synthase, **TM-02** was found to have a lower K_i_ (1.1 µM [0.51 µg/mL]) than **TM-03** (3.5 µM [1.63 µg/mL]) (Table 2). This ~3-times decrease in the concentration required to produce half of the inhibition reflects the lower MIC of **TM-02** vs. **TM-03** seen against all hypersusceptible strains (*S. aureus* Δ*hemB* or *E. coli* alt*lptD*, Table 1). This result also confirms what was observed by Lamontagne Boulet et al. (2018) [7], where the concentration of **TM-02** (*a.k.a.* FcM) needed to reduce *E. coli* ATP production (IC_50_) by 50% was 2.2 times lower than that of **TM-03** (*a.k.a.* Fcm).

These results demonstrate that both diastereoisomers are able to inhibit the activity of the ATP synthase target but that **TM-02** has a greater ability to do so. This also correlates with the stronger inhibitory activity of **TM-02** (vs. **TM-03**) against the hypersusceptible *S. aureus*Δ*hemB* and hyperpermeable *E. coli*-alt*lptD* strains. However, this does not explain why parental strains are equally susceptible to both diastereoisomers.

### 2.3. In Silico Docking Model Confirms the Greater Inhibition Potency of ***TM-02*** against the ATP Synthase Subunit c

An in silico docking model only using the ATP synthase subunit c was performed in order to corroborate the K_i_ experimentally measured with membrane vesicles. Two observations were made with the model. First, just like with the vesicle assay, **TM-02** showed a greater potency against the ATP synthase (vs. **TM-03**) with a modeled K_i_ of 5.1 µM and a free energy of −7.21 Kcal/mol. The modeled K_i_ for **TM-03** was 9.3 µM, which is 1.8 times higher than that predicted for **TM-02**, and had a free energy of −6.86 Kcal/mol (Table 2). For both compounds, the modeled K_i_ were higher than the experimental K_i_. This may be consequent to the fact that only the subunit c was considered in the in silico model while the entire the enzymatic complex within the membrane environment is present in the in vitro assay.

**Figure 2:**
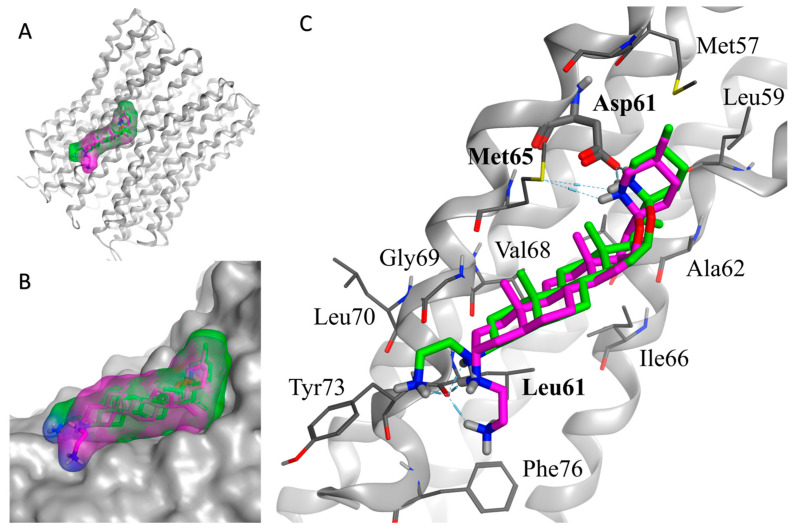
In silico docking model of **TM-02** (green) and **TM-03** (purple) to *E. coli* ATP synthase subunit c. (**A**) Low-energy conformation docked to the ATP synthase subunit c (grey). (**B**) Enlargement of the docking area showing the complementarity between the surfaces of the molecules and the subunit c. (**C**) Main interactions between the ligands (**TM-02** and **TM-03**) and the residues of ATP synthase. Oxygen, nitrogen and sulfur atoms are shown in red, blue and yellow, respectively. The *E. coli* ATP synthase subunit c structure was from the Protein Data Bank (PDB 6WNQ).

As shown in Figure 2, the overall binding mode of the two diastereoisomers (**TM-02** in green and **TM-03** in purple) is similar but has slight differences. The secondary amine of the F ring from the spiroaminoketal motif forms an important salt bridge with the aspartic acid 61 of the subunit c, which is essential for the ATP synthase energy production. This secondary amine also forms a hydrogen bond with the sulfur of methionine 65. In addition, a hydrogen bond network exists between the ethylenediamine at C3 and the carbonyl of the amide originating from leucine 72. The major difference arises from the orientation of the C-3 diamine of **TM-03**, which pushes the steroidal skeleton to move slightly away from the C subunit (Figure 2B), possibly reducing the strength of the van der Waals interactions with the subunit c, which is mainly composed of hydrophobic residues (Figure 2C).

This in silico model supports experimental K_i_ determinations derived from membrane vesicles and reinforces the fact that **TM-02** has a greater affinity for the target than **TM-03**.

### 2.4. Killing Kinetics of ***TM-02*** and ***TM-03***

**Figure 3:**
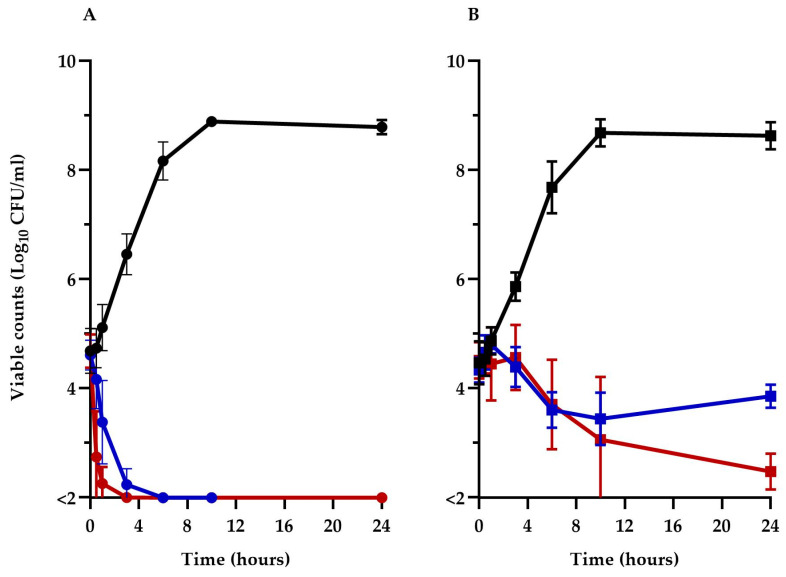
Kill kinetics of **TM-02** and **TM-03**. **TM-02** (blue) and **TM-03** (red) were used at 32 µg/mL (1× MIC) against (**A**) *E. coli* BW25113, and (**B**) *E. coli* BW25113Δ*atpE*. Growth without antibiotic is shown in black.

To better understand the impact of **TM-02** and **TM-03** on ATP synthase and their modes of action, the viability of the Keio collection *E. coli* BW25113Δ*atpE* mutant was compared to its parental strain, BW25113, over a 24 h period in the presence of 32 µg/mL (1× MIC) of these antibiotics. The prototypic *E. coli* BW25113 (Figure 3A) was rapidly killed (>3 log_10_ reduction in CFU/mL) by both **TM-02** and **TM-03** in less than 6 h, with **TM-03** showing a slightly more rapid bactericidal action. On the other hand, **TM-02** was revealed to be bacteriostatic against the Δ*atpE* mutant, only causing a loss of about 1 log_10_ CFU/mL after 24 h, whereas **TM-03** remained bactericidal (>3 log_10_ reduction in CFU/mL at 24 h), although its killing was slower than that seen for the parental strain.

These results clearly illustrate that the principal target of both diastereoisomers is the ATP synthase as they both lose significant antibiotic activity against the *atpE* deletion mutant. It is noteworthy that the killing mechanism of **TM-02**, which possesses the best K_i_, was completely abolished in the Δ*atpE* mutant. However, since **TM-02** and **TM-03** retained some antibacterial activity against the Δ*atpE* mutant, these results also indicate that there is a putative secondary mode of action for both molecules and that it would be more important for the activity of **TM-03** as it remains bactericidal against the Δ*atpE* mutant.

### 2.5. ROS Production Is Induced by Both Diastereoisomers

**Figure 4:**
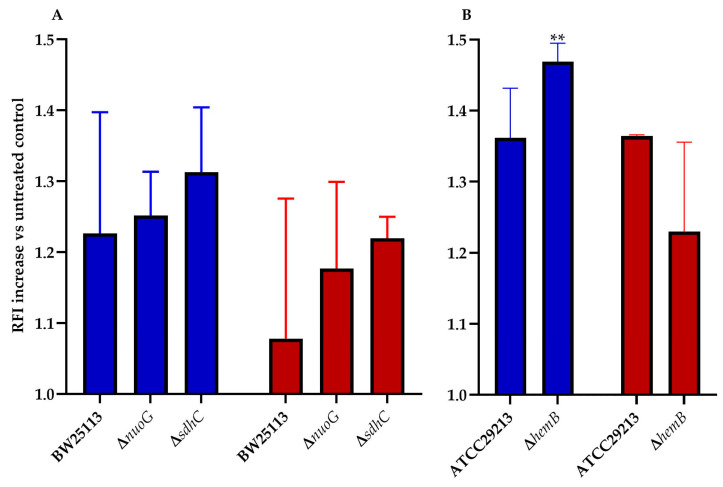
ROS production increase in electron transport chain mutants of *E. coli* and *S. aureus*. ROS production increase relative to the untreated parental strain (relative fluorescence intensity increase) for (**A**), *E. coli* BW25113, Δ*nuoG,* and Δ*sdhC* mutants, and (**B**), *S. aureus* ATCC29213 and Δ*hemB* mutants, in presence of either (blue) **TM-02** or (red) **TM-03** at 4× MIC. Statistical analyses were performed using a one-way Anova with a Dunnet’s multiple comparison test, comparing each condition to the no-antibiotic control (normalized to 1.0); ** *p* < 0.01.

ROS play a key role in the mechanism of action of all bactericidal antibiotics [12,13]. This statement holds true for the mode of action tomatidine [8], and, thus, ROS production in *E. coli* and *S. aureus* was measured for **TM-02** and **TM-03**. It was previously reported that mutations affecting the electron transport chain cause a sensitivity to ROS production in presence of antibiotics [14,15]. We, thus, explored ROS production resulting from exposure to **TM-02** and **TM-03** for a variety of electron transport chain mutants in *E. coli* and *S. aureus* (Figure 4). All strains (parental and mutants) produced 1.08 to 1.47 times more ROS in the presence of the antibiotics relative to untreated bacteria (RFI normalized to 1.0). Using two respiratory chain mutants from the *E. coli* Keio collection, *nuoG* and *sdhC*, we observed that there was a tendency for **TM-02** to generate more ROS production vs. **TM-03**, although this was not statistically significant due to experimental variations (Figure 4A). The only statistically significant increase in ROS production was obtained for the electron transport chain mutant *S. aureus*∆*hemB* upon exposure to **TM-02** (*p* < 0.01. Figure 4B).

This assay showed that, in a strain that is highly dependent on its ATP synthase and that has a defect in its respiratory chain like the SCV strain ∆*hemB*, **TM-02** leads to more production of ROS than **TM-03**.

### 2.6. ***TM-02*** and ***TM-03*** Affect Membrane Potential Differently

**Figure 5:**
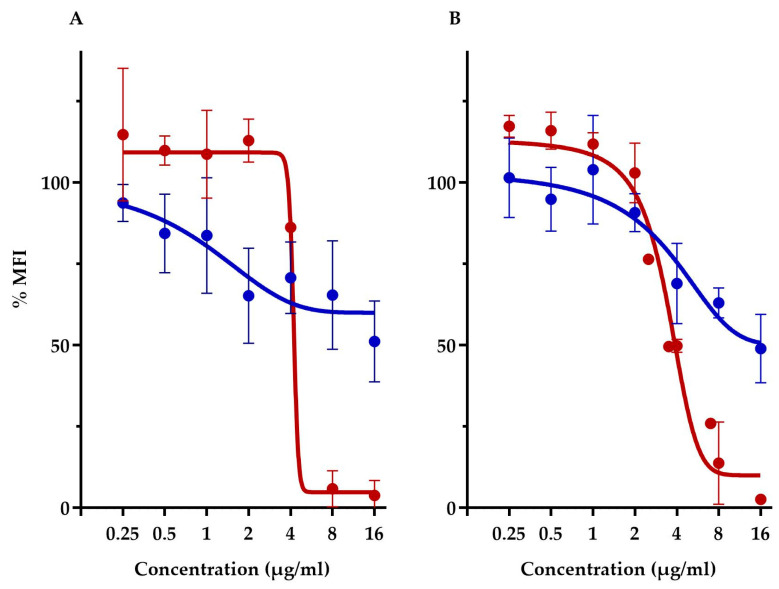
Relative membrane potential measurement in presence of **TM-02** and **TM-03**. Membrane potential (median fluorescence intensity) of bacterial strains in the presence of **TM-02** (blue) or **TM-03** (red): (**A**) *S. aureus* ATCC 29213 and (**B**) *E. coli* MC4100-alt*lptD*. The MFI was relative to that of each strain-untreated control.

This assay compared the membrane potential of *S. aureus* ATCC 29213 and *E. coli*-alt*lptD*. Note that we had to use, here, the hyperpermeable *E. coli* strain in order to allow the fluorescent probe to traverse the Gram-negative outer membrane barrier. First off, this assay showed that both diastereoisomers had vastly different membrane depolarization patterns. **TM-02** affects membrane potential very similarly to that previously observed with TO and *S. aureus* [8]. Both TO and **TM-02** produce a gradual membrane depolarization with increasing concentrations of antibiotics, and we showed here that this pattern is nearly identical for *E. coli* and *S. aureus* in the presence of **TM-02** (Figure 5). For both strains, **TM-02** reduced the membrane potential by 50% compared to that of the untreated control.

On the other hand, **TM-03** completely dissipated the membrane polarization of both *S. aureus* and *E. coli* at the same threshold concentration. There was no impact on membrane potential at concentrations below 4 µg/mL, which seemed to be the threshold concentration for depolarization as it dropped by about 50% compared to the untreated cells (Figure 5). At 8 µg/mL (doubling dilution scheme), the membrane potential was completely dissipated, reaching <10% of that of the untreated cells.

The drastically different membrane depolarization patterns observed for **TM-02** and **TM-03** are again indicative of different modes of action for the two diastereoisomers with **TM-03** showing, this time, a greater impact on bacterial membrane functionality.

### 2.7. ***TM-03*** Rigidifies the Bacterial Membrane

**Figure 6:**
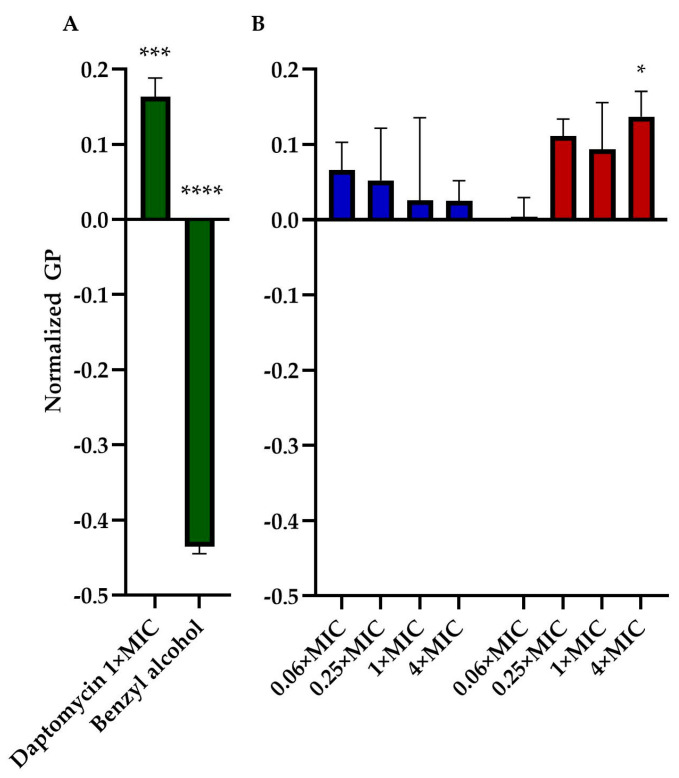
Membrane fluidity assessment. General polarization (GP), normalized to the untreated control of *B. subtilis* str.168, caused by (**A**) daptomycin (2 µg/mL) and Benzyl alcohol (1 mM) treatments (both in green); and by (**B**) **TM-02** (blue) and **TM-03** (red) at up to 4× MIC. Statistical analyses were performed using a two-way ANOVA with a Dunnett’s multiple comparison test, comparing each group to untreated control; * *p* < 0.01, *** *p* < 0.01, **** *p* < 0.01.

We assessed the impact of **TM-02** and **TM-03** on bacterial membrane fluidity using Laurdan microscopy. This assay was similarly used by Müller et al. (2016) [16] to measure the impact of daptomycin on bacterial membranes. We, thus, used daptomycin as a control agent that rigidifies membranes and benzyl alcohol as an agent that increases membrane fluidity. Moreover, for this assay, we used *B. subtilis*, a Gram-positive *Bacillales* which is larger in size than *S. aureus*, to facilitate microscopic analysis. The assay generated quite a bit of variation among replicates for each condition. There was a clear tendency, however, for **TM-03** to increase membrane rigidity compared to that provoked by **TM-02** (Figure 6). **TM-03** rigidified *B. subtilis* membranes from concentrations as low as 0.25× MIC up to 4× MIC (maximum tested) where the effect became statistically significant compared to the untreated control.

**Figure 7:**
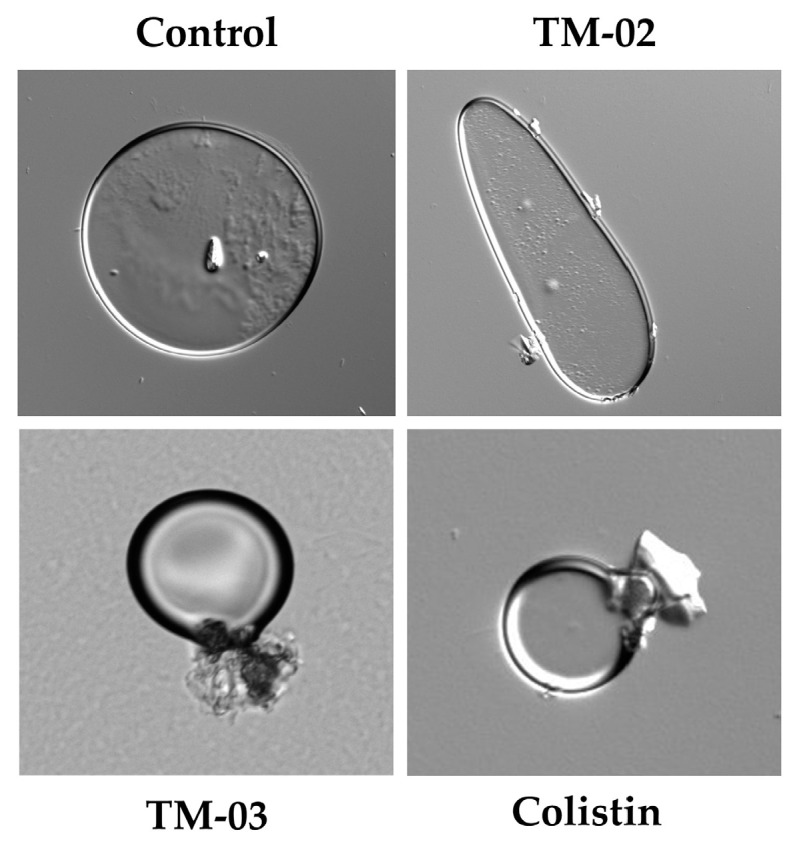
*E. coli* ATCC 25922 membrane vesicles in presence of antibiotics. Compounds were used at their MIC (**TM-02** and **TM-03** at 8 µg/mL; colistin at 0.5 µg/mL). Vesicles were treated for 30 min and imaged with differential interference contrast (DIC).

Based on these results, we wanted to see if effects on large membrane vesicles could be visible under a microscope. Therefore, the *E. coli* ATCC 25922 membrane vesicles that we had prepared for ATP synthase assays were exposed 30 min to **TM-02** or **TM-03** at their MIC before microscopic examination (Figure 7). We also used the antibiotic colistin, known for its cytoplasmic membrane disrupting action, as a comparator drug. Microscopic observations showed that **TM-02** merely pinched or affected the spherical shape of the vesicles, whereas **TM-03** strongly damaged membrane vesicles, similarly to that seen for colistin (Figure 7).

These results imply once again slightly different mechanisms of action for **TM-02** and **TM-03**. The latter would have a greater tendency to affect bacterial membranes.

### 2.8. ***TM-03*** Has a Longer Post-Antibiotic Effect (PAE)

To examine if the diastereoisomers have a persisting effect on bacterial cells after a short antibiotic exposure, we proceeded to measure the PAE, which is a value normally used to help in determining dosing regimens in the clinical setting. We measured the PAE of **TM-02** and **TM-03** for *S. aureus* and *E. coli* (Table 3 and Figure S1). **TM-02** had a PAE of 1.14 h and **TM-03** had a PAE of 2.09 h at 4× MIC for *S. aureus*. The most important difference was observed at 1× MIC with a PAE of 0.06 h and 0.73 h (*p* < 0.0001), for **TM-02** and **TM-03**, respectively. For *E. coli*, the PAE of **TM-02** was 0.18 h and 2.37 h at 1× and 4× MIC. For **TM-03**, *E. coli* had a PAE of 0.47 h at 1× MIC and we could not observe regrowth at 4× MIC (*p* < 0.0001). There was no PAE at 0.5× MIC for either *S. aureus* or *E. coli*.

These results are consistent with the possibility that there might be an accumulation of compounds in bacterial membranes and that this may be particularly true for **TM-03**, showing a long-lasting inhibitory effect on growth after removal of the compound from the medium. This is observed at a lower concentration for *S. aureus* (8 vs. 32 µg/mL for *E. coli*), possibly because of the additional outer membrane limiting the access to the cytoplasmic membrane and the cellular target in the Gram-negative bacterium.

### 2.9. No Resistance Arose from the Exposition to Sub-Inhibitory Concentrations of ***TM-02*** or ***TM-03***

**Figure 8:**
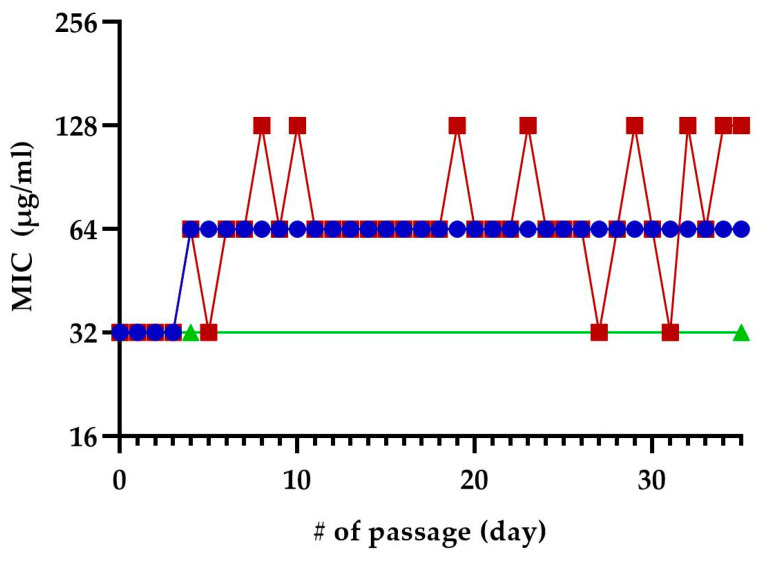
Selection of resistance using sequential passage on sub-inhibitory concentration of **TM-02** and **TM-03**. *E. coli* MC4100 was sequentially exposed to sub-inhibitory concentrations of **TM-02** (blue) or **TM-03** (red) for a total of 35 passages. Passage numbers (#) also indicate days. The increase in MIC observed during the passages could not be reproduced from neither of the three colonies isolated from the passages 4, 31, and 35, and the green line represents the confirmed MIC of those isolates.

We exposed *E. coli* MC4100 to sub-inhibitory concentrations of **TM-02** or **TM-03** for 35 passages to see if resistance to these molecules could develop after repeated exposures (Figure 8). At the fifth passage, both **TM-02** and **TM-03** MIC went from 32 µg/mL to 64 µg/mL. For **TM-02**, the MIC was then unchanged for the 30 subsequent passages. **TM-03**, on the other hand, had a varying MIC, oscillating between 32 µg/mL (original MIC) and 128 µg/mL (4× MIC). To verify these apparent changes in MIC, three colonies were isolated from each of the passages 4, 31, and 35. These passages corresponded to the passage before the first increase in MIC, the passage where **TM-03** MIC went from 64 µg/mL to 32 µg/mL, and the passage where **TM-03** MIC was 128 µg/mL, respectively. When the MIC of these isolates where tested, there was no increase in MIC, with the MIC of both **TM-02** and **TM-03** remaining at 32 µg/mL.

These results suggest that the resistance acquisition rate towards these antibiotics is low.

### 2.10. In Vivo Efficacy of ***TM-02***

**Figure 9:**
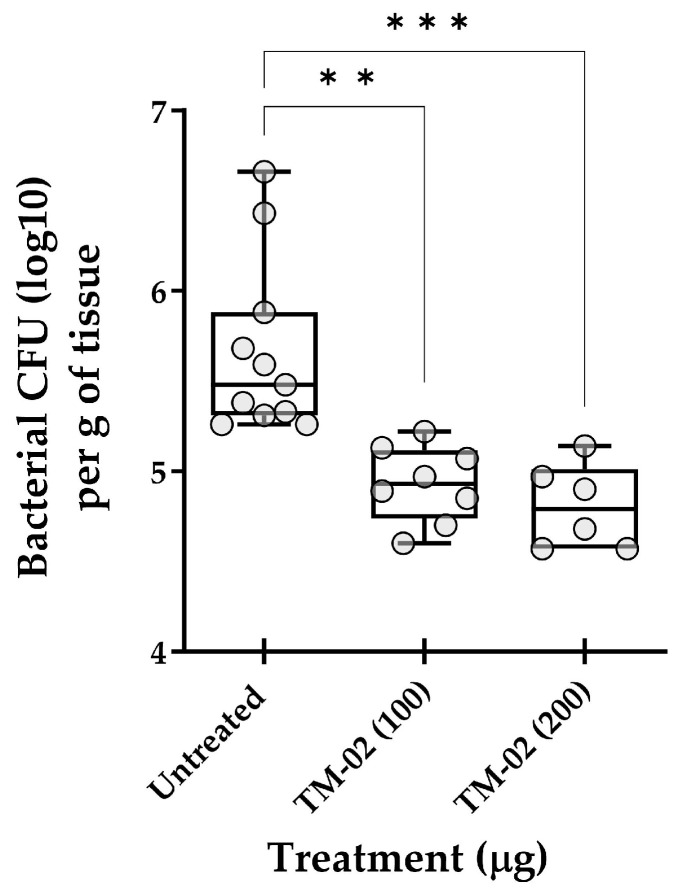
Treatment of pulmonary *S. aureus* SCV infections in mice with **TM-02**. Mice were inoculated intratracheally with ~5 × 10^7^ CFU of strain NewbouldΔ*hemB* and were treated by the same route 6 h later using **TM-02** at the indicated doses. Bacterial loads (CFU) in the lungs of animals were evaluated 16 h post-treatment. Each circle on the graph represents the bacterial load measured in each mouse. For each dataset, the boxplot displays the minimum value, first quartile, median, third quartile, and maximum value. The statistical significance of CFU reduction for treated animals compared to untreated controls (diluent only) was determined by the non-parametric Kruskal–Wallis ANOVA test, followed by the Dunn’s test for multiple comparisons. (**, *p* < 0.01; ***, *p* < 0.001).

As an ultimate test, we explored the in vivo efficacy of **TM-02**, the most active and specific C3-diastereoisomer, for the treatment of mouse pulmonary infections caused by *S. aureus* NewbouldΔ*hemB*. Figure 9 reports the in vivo data and shows that **TM-02** could significantly reduce the bacterial loads in lungs in a dose-dependent manner. These results show that **TM-02** is well-tolerated in the animals and that it is active in the lung environment when administered intratracheally.

## 3. Discussion

TO is an efficient antibiotic molecule against the SCV phenotype of *S. aureus* [6] but lacks efficacy against WT strains. Modifying its C3 hydroxyl to an ethane-1,2-diamine (Figure 1) broadens its antibiotic spectrum and efficacy against a larger array of bacteria, while retaining its strong anti-SCV activity. Furthermore, it was found that **TM-02** and **TM-03** target the bacterial ATP synthase subunit c [7] and that such C3 ethane-1,2-diamine compounds work in synergy with aminoglycosides similarly to that reported for TO [11].

As reported in the present study, **TM-02** presented more activity (lower MIC) against *S. aureus* SCV strains (known to be hypersusceptible to ATP synthase inhibitors [8]), compared to **TM-03**. Moreover, TO-resistant SCV strains (TO MIC > 128 µg/mL) did not exhibit a complete resistance to **TM-02**, showing an MIC of 2 µg/mL while **TM-03** showed an MIC similar to that measured for WT strains (8 µg/mL). Similarly, **TM-02** was more active than **TM-03** against *E. coli* hyperpermeable strains (Table 1). Both **TM-02** and **TM-03** also significantly lost activity against *S. aureus* SCVs or hyperpermeable *E. coli* overexpressing the ATP synthase subunit c (*atpE*). These results suggest that, although both diastereoisomers can affect the bacterial ATP synthase, **TM-02** is more potent than **TM-03** toward the enzyme as supported by our in silico and experimental K_i_ data (Table 2 and Figure 2). On the other hand, the intriguing observations that both **TM-02** and **TM-03** had the same activity against the WT *S. aureus* (MIC of 8 µg/mL for both) and *E. coli* strains (MIC of 32 µg/mL), and that the loss of the ATP synthase subunit c in *E. coli* only affected the bactericidal activity of **TM-02** (Figure 3), raised the question as to whether there was a common secondary mode of action for these compounds in addition to their ability to inhibit ATP synthase. The work presented here attempted to answer that question.

It was previously found that **TM-02** reduces ATP generation more efficiently than **TM-03** [7]. We took it a step further in this work and analyzed the enzyme inhibition kinetics to learn more about the primary mechanism of action of these diastereoisomers. With a nearly 3-times lower inhibition constant (K_i_), **TM-02** was confirmed to be a more potent bacterial ATP synthase inhibitor. This finding is important because it explains why **TM-02** is more effective against SCV strains that are known to be hypersusceptible to ATP synthase inhibitors [8]. Furthermore, using the *atpE* deletion mutation of *E. coli* BW25113, we saw that both **TM-02** and **TM-03** were less effective at killing the mutant (Figure 3). However, although much slower in its action, **TM-03** was able to retain its bactericidal activity while **TM-02** became bacteriostatic. The fact that **TM-02** becomes bacteriostatic in the absence of the ATP synthase implies that its killing mechanism against the wild-type strain is indeed dependent on the inhibition of the enzyme and that the marked difference observed in the bactericidal patterns of **TM-02** and **TM-03** may be due to their respective affinity to the target. These results also demonstrated that there was at least another target, other than the ATP synthase, allowing the antibiotics to either inhibit (**TM-02**) or kill (**TM-03**) the *E. coli atpE* deletion mutant. Below, we discuss the possible contribution of ROS in the bactericidal action of **TM-02** and perhaps the contribution of stronger membrane perturbations in the bactericidal activity of **TM-03**.

ROS play a key role in the activity of ATP synthase inhibitors. These molecules are by-products naturally occurring during aerobic respiration [17], but the overproduction of ROS is often a consequence of the presence of bactericidal antibiotics [13]. **TM-02** and **TM-03** data show that both antibiotics are bactericidal and lead to considerable ROS production in WT *E. coli* and *S. aureus*. However, with **TM-02**, considerably more ROS is produced in the SCV phenotype of *S. aureus* (Figure 4). As SCVs are generally mutated in their electron transport chain, we sought to investigate whether similar mutations in *E. coli* could also lead to more ROS production. Results show that electron transport chain mutants tended to produce more ROS in the presence of **TM-02**. An increase was also noted in the presence of **TM-03**, but the ROS levels produced were lower than the prototypical strain in the presence of **TM-02**. These results illustrate the particular importance of the affinity to the target, the bacterial ATP synthase, in the bactericidal mechanism of action of **TM-02**, but also that an impaired electron transport chain can indeed accentuate ROS production.

As previously stated, *S. aureus* SCVs are more vulnerable to ATP synthase inhibitors such as TO [8]. This is because their membrane potential is already weak and that ATP synthase inhibitors reduce it under a minimal threshold. This effect of TO on membrane potential was only observed for *S. aureus*, with no effect in *E. coli,* as this molecule is very specific to *Bacillales* ATP synthase [6,7]. With TO C3-analogs now able to target both *E. coli* and *S. aureus*, it was possible to explore the effect of these antibiotics on the membrane potential of these species. Our results showed that increasing concentrations of **TM-02** gradually reduced the bacterial membrane potential for *E. coli* and *S. aureus*, but that the **TM-03** effect was much more drastic with a complete depolarization of membranes when the antibiotic concentration reached a certain threshold (Figure 5). Such an effect suggested that **TM-03** altered membrane functionality differently than **TM-03**, and this needed to be explored further.

We thus looked at the possibility that **TM-02** and **TM-03** modified the fluidity of the membrane, since they both share a steroidal scaffold that could interact with biological membranes [18]. Müller et al. (2016) [16] used Laurdan as a reporter for membrane fluidity and were able to demonstrate that daptomycin causes the rigidification of bacterial membranes. Here, the membrane fluidity of *B. subtilis* was shown to be statistically unaffected by **TM-02**, whereas, at concentrations as low as a fourth of the MIC, **TM-03** tended to rigidify the membranes (Figure 6). This finding matched the depolarizing effect of **TM-03** on the cytoplasmic membrane of *S. aureus* as discussed above. The strong effect of **TM-03** on biological membranes was also actually observed by the microscopic examination of *E. coli* membrane vesicles exposed to the compound and was comparable to that caused by the cytoplasmic membrane-disrupting antibiotic colistin [19] (Figure 7). Instead, treatment by **TM-02** affected the spherical shape of membrane vesicles, and, interestingly, this observation was reminiscent of that observed after purified mitochondrial ATP synthase dimers self-assemble into liposomes where they induce membrane curvature [20]. It will be interesting to see if **TM-02** can indeed cause some rearrangements of bacterial ATP synthase complexes in future studies. Besides, because the effects of **TM-03** could be due to an accumulation of the antibiotic into the bacterial membrane, we looked for the duration of the post-antibiotic effect in the hope to find yet another indication of this. What we could conclude from this assay was that **TM-03** had indeed a longer-lasting effect on bacteria than **TM-02** after its removal from the medium. Although the longer PAE of **TM-03** could be explained by a greater accumulation in the membranes, this hypothesis needs to be confirmed by further work.

In this work, we collected evidence that both **TM-02** and **TM-03** inhibit the bacterial ATP synthase, with **TM-02** being the most potent. As shown before, a reduction of ATP production in *S. aureus* can lead to a reduction of macromolecular syntheses [6] and a reduced expression of virulence factors [9]. This can also lead to a reduction of the membrane potential and generation of ROS, which can also contribute to the activity of **TM-02** and **TM-03**. Data also show that either the overexpression or deletion of the ATP synthase subunit c (AtpE, the specific target of TO [7]) specifically reduces the antibiotic activity of these diastereoisomers, although some residual activity remains. We propose that this residual or secondary mode of action is linked to their interaction with the bacterial membrane, with **TM-03** being the most potent on this front. This assumption is based on the more pronounced membrane depolarization, reduction of membrane fluidity, and breaks in membrane vesicles occasioned by **TM-03**.

More work is needed to determine the precise secondary mechanism of action, and how the spatial position of the C3 ethane-1,2-diamine influences the interaction of these compounds with the bacterial membranes. **TM-02** has a higher affinity for the ATP synthase, which seems to be responsible for its bactericidal action while the contribution of the secondary mode of action seems more limited. **TM-03** has a lower affinity for the enzyme, resulting in a decreased activity against the hypersusceptible SCV strains of *S. aureus* (Δ*hemB*) and the hyperpermeable *E. coli* (alt*lptD*), but is as efficient as **TM-02** against WT strains presumably because of its stronger interaction with the membranes. Interestingly, this dual mode of action may limit the emergence of resistance. This hypothesis is reflected in the fact that neither **TM-02** nor **TM-03** were able to generate *E. coli*-resistant mutants after 35 passages in presence of sub-MICs of these compounds (Figure 8), as was shown before for **TM-02** and *S. aureus* [7]. The low frequency of resistance development and sensibilization of *S. aureus* to antimicrobial peptides [21] and aminoglycosides [22] further support the development of ATP synthase inhibitors as an antibiotic class. **TM-02** treatment efficacy against a lung infection induced by a *S. aureus* SCV strain in mice (Figure 9) also represented an important step in the antibiotic development process by showing tolerability in animals.

## 4. Materials and Methods

### 4.1. Enantioselective Synthesis of ***TM-02***

**Scheme 1:**
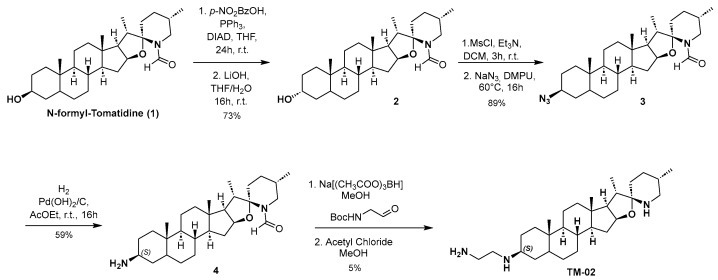
Enantioselective synthesis of **TM-02**.

Compounds **TM-02** and **TM-03** can be directly obtained as a diastereomeric mixture at the C-3 position by reductive amination from the corresponding ketone, followed by purification using preparative HPLC-MS as previously published [23]. However, to confirm the absolute configuration at the C-3 position, an enantioselective synthesis of **TM-02** was developed as described in Scheme 1.

N-formyl Tomatidine **1** was synthesized following literature reported by Chagnon et al. [10]. From **1**, the stereochemistry inversion of the C-3 alcohol was performed in a two-step procedure through a Mitsunobu reaction using 4-nitrobenzoic acid followed by hydrolysis of the corresponding ester to give compound **2**. Subsequently, the desired configuration at C3 was obtained by mesylation of the alcohol, and then nucleophilic substitution with sodium azide with excellent yield. A catalytic hydrogenation with palladium hydroxide and hydrogen leads to amine **4** with the (S) configuration. The secondary amine is then alkylated by reductive amination using N-Boc-2-aminoacetaldehyde in presence of sodium triacetoxyborohydride. Finally, **TM-02** was obtained by treating **4** with a solution of acetyl chloride in anhydrous methanol and purified by preparative HPLC-MS.

Enantioselective synthesis of **TM-02** and compound characterization are provided in Supplemental Figure S2.

### 4.2. Strains and Growth Conditions

Some of the parental strains used in this study were *S. aureus* ATCC 29213 and *E. coli* MC4100. The ATP synthase inhibitor hypersusceptible SCV strains were *S. aureus* ATCC 29213∆*hemB* and *S. aureus* Newbould∆*hemB*. Those stable SCV strains were constructed by disrupting the *hemB* gene involved in hemin biosynthesis by homologous recombination using *ermA* cassette [24,25]. Also, *S. aureus* strain Newbould∆*hemB_atpE* (SaR1-1) carrying a point mutation in AtpE (A17S) leading to tomatidine resistance was previously described [7]. In addition, we used a *E. coli* hyperpermeable strain (*E. coli* MC4100-alt*lptD*), previously named imp4213 [26], having an altered *lptD* gene with a short in-frame deletion and previously named imp4213 [26]. Other strains used in this study were *B. subtilis* ATCC 23857 for the microscopic analysis and *E. coli* ATCC 25922 for the ATP production and inhibition assays and *E. coli* BW25113Δ*atpE* from the Keio collection [27] for the antibiotic kill kinetics assays. Bacteria were maintained in tryptic soy broth (TSB) or on tryptic soy agar (TSA) at 35 °C or 37 °C for *E. coli* strains.

### 4.3. Generation of atpE Overexpression Strains

*E. coli* MC4100-alt*lptD* was used to insert pAC027_ColE1 containing its *atpE* gene. pAC027_ColE1 [28]. Briefly, *E. coli* MC4100 *atpE* gene was amplified using these primers which added a sequence for the enzymatic digestion with EcoRI and NotI:Fwd AGG ACG CAC TGACCG AAT TCA TAA AGA GGA AGG TAC CAT GGA AAA CCT GAA TAT GGA and rev TAA AAG TTG ATT TGC GGC CGC CTA CGC GAC AGC GAA CAT CA. Afterwards, plasmid and insert were cut using EcoRI and NotI (New England Biolabs [NEB] Inc., Pickering, ON, Canada), according to manufacturer’s protocol. The ligation was then performed overnight at 16 °C. Transformant were selected on ampicillin-containing TSA plates. In addition, the construction of a *S. aureus* SCV strain overproducing the *atpE* gene (NewbouldΔ*hemB* pCN36-*atpE*) was previously described [7].

### 4.4. Antibiotic Susceptibility Testing

Antibiotic MICs were determined by a broth microdilution technique according to the recommendations of the Clinical and Laboratory Standards Institute (CLSI) 2018 [29], using the cation-adjusted Mueller–Hinton (CAMHB) broth or TSB to better sustain the growth of *S. aureus* SCV strains. Microtiter plates were incubated for 20 h under aerobic conditions at 35 °C or 37 °C for *E. coli* strains. Antibiotics in CAMHB were diluted by two-fold serial dilutions, and an equal volume of the ~10^6^ colony-forming units (CFU)/mL inoculum was added to each well. Plates were incubated for 18–24 h at 35 °C for staphylococci or 37 °C for *E. coli*. Vancomycin, gentamicin, and erythromycin were always used as control drugs and the quality-control strains *S. aureus* ATCC 29213 and *E. coli* ATCC 25922 were used in all assays. The MIC was defined as the lowest concentration of antibiotic yielding no visible growth.

### 4.5. Bacterial ATP Synthase Inhibition Assay

(i) Preparation of *E. coli* membrane vesicles. Membrane vesicles containing the bacterial ATP synthase were prepared as previously described [23]. Briefly, *E. coli* ATCC 25922 membrane vesicles were prepared from a 1.2 L broth culture grown to an A_600_ of 0.8–0.9. Bacteria were collected by centrifugation at 4000× *g* for 20 min at 4 °C. Pellets were frozen at −80 °C for 24 h and then thawed on ice for 30 min in a lysis buffer consisting of 50 mM MOPS, 10 mM MgCl_2_, 10% glycerol, 100 µg/mL lysozyme, 8 µg/mL DNAse, and 1 mM PMSF. Bacteria were lysed in a French pressure cell at 18,000 lb/in^2^. The bacterial lysate was then centrifuged at 10,000× *g* at 4 °C for 20 min to remove unbroken cells before ultracentrifugation at 150,000× *g* for 40 min at 4 °C to collect membrane vesicles in MOPS-MgCl_2_ with 10% glycerol. Aliquots were kept at −80 °C. Protein concentration was estimated using Micro BCA protein assay kit (Thermo Fisher, Rockford, IL, USA) and using bovine serum albumin as a standard.
(ii) Enzyme kinetics and Ki determination. For these assays, 22.5 µg of membrane vesicles were mixed with a 10 mM MOPS-MgCl_2_ buffer. Inhibitors were then added at concentrations ranging from 0 to 12 µM. To start the reaction, 2.5 mM NADH and 1 mM ADP were added. The reaction was stopped after 10 min with 2 mM EDTA containing 1% trichloroacetic acid. Each sample was diluted 1:20 in Tris acetate buffer and luminescence was measured using ATPlite 1-step Luminescence Assay system (Perkin Elmer, Woodbridge, ON, Canada) in a LumiStar OPTIMA microplate reader. Data were analyzed using the method of Burlingham et al., 2003 [30]. Briefly, the inverse of the luminescence production rate (s/RLU) was plotted against the inhibitor concentration. The y-intercept value was then divided by the slope. To obtain the K_i_, the value previously calculated is corrected with the K_m_.

### 4.6. In Silico Docking Model

The AutoDock4 4.2.6 program was used to perform the docking analysis. *E. coli* ATP synthase crystal structure (PDB 6WNQ) was obtained from Protein Data Bank website (rscb.org), accessed on 15 October 2023, and only subunit c was kept for the analysis. To prepare the protein for docking, water molecules were removed, polar hydrogens were added, and Kollman charges were calculated. Of all the docking possibilities resulting from the analysis, the best-fitting orientation, according to previous docking results obtained [23], were considered. Results were reported as free energy and estimated K_i_. Images were produced and analyzed using the Molecular Operating Environment (MOE), version 2022.02, of the Chemical Computing Group ULC (Montreal, QC, Canada).

### 4.7. Bactericidal Activity

The bactericidal activity of antibiotics over time was measured in CAMHB under aerobic conditions with agitation (225 rpm), based on CLSI recommendations [31]. The inoculum was ~10^5^–10^6^ CFU/mL and cells were grown at 37 °C. Over a 24 h period, cultures with or without antibiotics were sampled and bacteria were serially diluted before plating on TSA for CFU determinations. A killing of ≥99.9% of the initial inoculum in ≤24 h indicated bactericidal activity.

### 4.8. Reactive Oxygen Species (ROS)

The method used for quantification of ROS was inspired by a published method [13], as previously described [8]. Bacteria freshly grown in TSB were suspended to an A_600nm_ of 0.1 in presence of 10 μM 2′,7′-dichlorodihydrofluorescein diacetate (H_2_DCFDA) (ThermoFisher, Rockford, IL, USA). Bacteria were incubated for 1 h. Then, bacteria were washed and suspended in TSB before distribution in a black, flat-bottom 96-well plate. Bacterial strains were exposed to either 1× or 4× MIC (in triplicate), and placed in a TECAN Genios Fluorescence plate reader set with the fluorescein settings. The peak fluorescence value from readings taken every 15 min at 9 positions per well during 13 h was recorded. Data were expressed as a relative fluorescence intensity (RFI) after background fluorescence was subtracted. An RFI of 1.0 was attributed to the control condition without antibiotics.

### 4.9. Measurement of Membrane Potential

The method used to measure the membrane potential was previously described [8]. Briefly, the method used the fluorescent dye indicator 3,3′-diethyloxacarbocyanine iodide (DiOC_2_) (ThermoFisher, Rockford, IL, USA). Bacteria previously grown on TSA plates were suspended in TSB with various concentrations of either **TM-02** or **TM-03** for 1 h. Cells were then washed and resuspended in PBS before addition of 3 mM DiOC_2_ for an incubation of 30 min at 35 °C. Bacterial cells were analyzed with the red (FL3-H) and green (FL1-H) channels of a FACScalibur instrument using a 488 nm laser. The FCS Express 6 Flow research program allowed data analyses. A higher membrane potential was represented by a higher red intensity (MFI) and results were expressed relatively to the MFI determined for the untreated control (MFI of 100%) [8].

### 4.10. Laurdan Microscopy

The membrane general polarization (GP) was measured using the Laurdan (1-[6-(Dimethylamino) naphthalen-2-yl] dodecan-1-one) (ThermoFisher, Rockford, IL, USA). Briefly, bacteria were incubated overnight on TSA. Bacteria were diluted 1:100 and grown up to a A_600_ of 0.4. Afterwards, 10 µM Laurdan was added for 10 min. Bacteria were washed 4 times in preheated microtubes. Bacteria were then exposed to either **TM-02** or **TM-03** at various concentrations. Images were taken on a Zeiss observer Z1 with an excitation wavelength of 350 nm and emission of 440 nm and 520 nm. The images were analyzed using ImageJ and GP was calculated using this formula [16]:

(I_440_ − I_520_)/(I_440_ + I_520_)

### 4.11. Membrane Vesicles Microscopy

Membrane vesicles were prepared as previously stated. They were then exposed to either **TM-02** (8 µg/mL), **TM-03** (8 µg/mL), or colistin (0.5 µg/mL) for 30 min. Membrane vesicles were imaged using a Zeiss observer Z1 with a differential interference contrast (DIC) method. The images were treated using ImageJ (v1.54f).

### 4.12. Post-Antibiotic Effect

The determination of the post-antibiotic effect (PAE) of **TM-02** and **TM-03** on *S. aureus* and *E. coli* was performed by using the method of Craig and Gudmundsson, 1991 [32]. Bacteria (~10^7^ CFU/mL) were first exposed for 2 h to the antibiotic in CAMHB at 0.5×, 1×, or 4× MIC. Bacteria were then diluted 1000× in a pre-warmed broth without antibiotic before allowing growth to resume. At several points in time, growth was monitored by CFU plate counting to measure any PAE. The PAE was defined as the period of residual inhibition of bacterial growth after antibiotic removal from the media. The PAE was measured with the following equation:

PAE = T − C

where T is the time required for the test culture to demonstrate a tenfold (1 log_10_) increase in CFU per ml immediately after removal of the antibiotic and C is the time required for the count of CFU in the untreated control culture to also increase tenfold above the count observed after the dilution.

### 4.13. Serial Passage of E. coli MC4100 on Sub-MIC of ***TM-02*** and ***TM-03***

In an attempt to generate **TM-02**- and **TM-03**-resistant mutants, we performed serial passage of *E. coli* MC4100 (35 passages of 24 h each) in a series of 2-fold dilutions of antibiotics (ranging from 0.5 to 256 µg/mL in CAMHB) in 96-well plates. At each passage, the MIC was determined, and the well representing 0.5× MIC was diluted in fresh broth and used to inoculate (~10^6^ CFU/mL) a new series of **TM-02** and **TM-03** dilutions for the next passage. At each passage, the 0.5 x MIC well was also plated on TSA, and, the next day, three isolates were collected and frozen before determining their precise MIC.

### 4.14. Mouse Model of Pulmonary Infection

The model of infection was performed as already described [33] with only a few modifications. Briefly, *S. aureus* Newbould∆*hemB* colonies grown on Brain Heart Infusion agar plates for 18 h were used to prepare a bacterial suspension in endotoxin-free PBS (Sigma-Aldrich, Oakville, ON, Canada). Bacteria were washed in PBS and diluted to the number of CFU required for infection using McFarland standards. The inoculum count was confirmed by serial dilutions and growth on agar plates after incubation at 35 °C. For the infection, CD-1 female mice (22–24 g, Charles River Laboratories, Saint-Constant, QC, Canada) were anesthetized and inoculated by intratracheal administration of 50 µL of approx. 5 × 10^7^ CFU using a 250 µL glass syringe and a bent 24 G feeding needle (Fine Science Tools, North Vancouver, BC, Canada). The inoculation procedure was performed using an otoscope and a speculum (model 21700, Welch Allyn, Mississauga, ON, Canada). The feeding needle was inserted into the trachea (~1 cm) before a slow instillation of the inoculum. At 6 h post-inoculation, mice were treated using the same exact procedure for instillation of 100 µg or 200 µg of compound **TM-02** in a 50 µL volume. After 16 h, mice were humanely euthanized and both lungs were collected and homogenized in 1.5 mL PBS using a Polytron homogenizer. The diluent used to solubilize the antibacterial compound, as well as the diluent used as control for untreated animals, was PBS containing 10% DMSO. The bacterial count (CFU per g of tissue) was determined by plating serial dilutions of gland homogenates on agar plates. Two independent experimental infections were carried out and data were combined.

### 4.15. Statistical Analysis

Statistical analyses were carried out with the GraphPad Prism Software (v.9.3.1). Statistical tests used for each experiment are specified in the legends of associated figures.

## 5. Patents

The TM compounds described in this article are the subject of several issued and pending patent applications, including US patent no. 10,716,797 B2 (issued 21 July 2020).

## Figures and Tables

**Table 1:** Antibiotic susceptibility profiles for bacterial strains used in this study.

Bacterial Strain	Characteristic	Antibiotic MIC (µg/mL)				
		TO	TM-02	TM-03	GEN	VAN
Gram-positive						
*S. aureus*						
ATCC 29213	WT	>128	8	8	0.25	0.5
ATCC 29213Δ*hemB*	SCV	0.06	0.5	4	8	1
Newbould	WT	>128	4	4	0.25	0.5
NewbouldΔ*hemB*	SCV	0.06	0.06	2	8	1
NewbouldΔ*hemB* pCN36-*atpE*	SCV, overproducing AtpE	>128	8	64	8	1
NewbouldΔ*hemB_atpE*	SCV, AtpE mutation A17S	>128	2	8	8	1
*B. subtilis* str. 168	WT	>128	8	8	0.12	-
Gram-negative						
*E. coli*						
ATCC 25922	WT	>128	32	32	-	-
MC4100	WT	>128	32	32	0.5	>128
MC4100-alt*lptD*	Hyperpermeable	>128	1	8	0.25	0.25
MC4100-alt*lptD* pAC027_ColE1-*atpE*	Hyperpermeable, overproducing AtpE	>128	64	64	-	-
BW25113	WT	>128	32	32	-	-
BW25113Δ*atpE*	*atpE* transposon mutant	>128	32	32	-	-

Abbreviations: ATCC, American Type Culture Collection; WT, wild-type strain; SCV, small colony variant; AtpE, ATP synthase subunit c; reference antibiotic GEN, gentamicin; VAN, vancomycin. The > sign represents the highest concentration tested; the “-“ sign means that it was not tested for that bacterial strain.

**Table 2:** Inhibition constant (K_i_) of **TM-02** and **TM-03**.

Compound	Free Energy Kcal/mol	Ki (µM) In Silico	Ki (µM) In Vitro	Km (µM) In Vitro
**TM-02**	−7.21	5.1	1.1	0.0985
**TM-03**	−6.86	9.3	3.5	

**Table 3:** Post-antibiotic effect (PAE) of **TM-02** and **TM-03** on *S. aureus* and *E. coli*.

	PAE (Hours) at the Indicated Concentration		
Strain and Compound	0.5× MIC	1× MIC	4× MIC
*S. aureus* ATCC 29213			
Ciprofloxacin	0.38 ± 0.53	0.78 ± 0.23	1.11 ± 0.42
**TM-02**	−0.17 ± 0.05	0.06 ± 0.06	1.14 ± 0.87
**TM-03**	−0.13 ± 0.03	0.73 ± 0.13	2.09 ± 0.94
*E. coli* MC4100			
Ciprofloxacin	1.91 ± 0.92	3.04 ± 1.95	4.09 ± 2.28
**TM-02**	0.09 ± 0.04	0.18 ± 0.13	2.37 ± 0.51
**TM-03**	0.10 ± 0.10	0.47 ± 0.34	no regrowth

## Data Availability

Data are contained within the article and Supplementary Materials.

## Supplementary Materials

The following supporting information can be downloaded at: https://www.mdpi.com/article/10.3390/molecules29020343/s1, Figure S1: Post-antibiotic effect of **TM-02** and **TM-03** against *S. aureus* and *E. coli*. Figure S2: Enantioselective synthesis of **TM-02** and compound characterization.
