# Supplementary material for: Mode of Antibacterial Action of Tomatidine C3-Diastereoisomers
Source: Molecules. 2024 Jan 10;29(2):343. doi: 10.3390/molecules29020343 (PMC10821064; doi:10.3390/molecules29020343)
Supplement: Supplementary file 1 [file molecules-29-00343-s001.zip › molecules-2734258-supplementary.pdf]

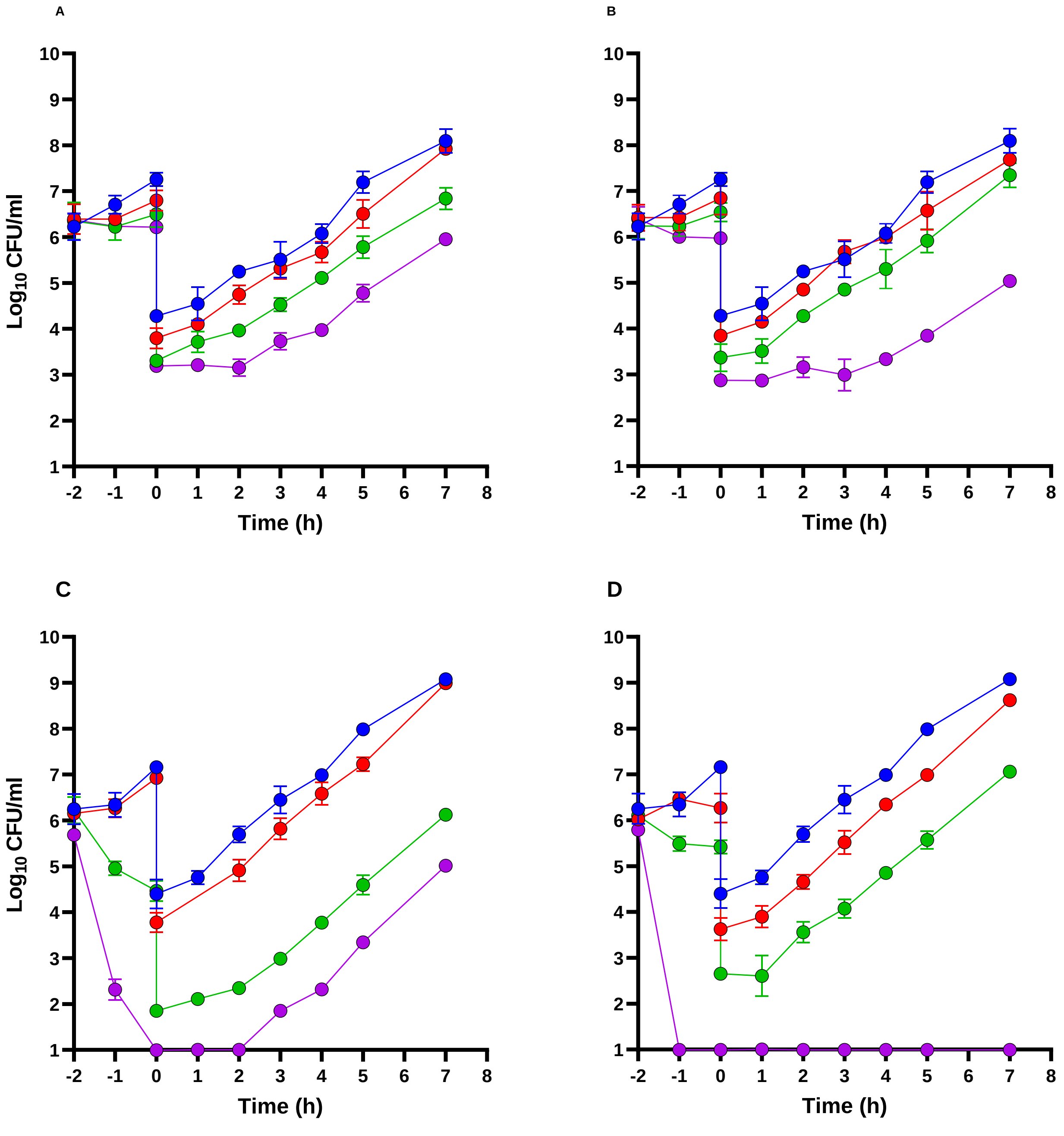

Figure S1: **Post-antibiotic effect of test compounds. (A)** *S. aureus* ATCC 29213 against TM-02, **(B)** *S. aureus* ATCC 29213 against TM-03, **(C)** *E. coli* MC4100 against TM-02 and **(D)** *E. coli* MC4100 against TM-03. Concentrations used range from no antibiotic (blue), 0.5 x MIC (orange), 1 x MIC (green) to 4 x MIC (purple). Results are reported as the time it took to increase the bacterial count by 1 log following T = 0 h.

Figure S2: Enantioselective synthesis of TM-02 and compound characterization.

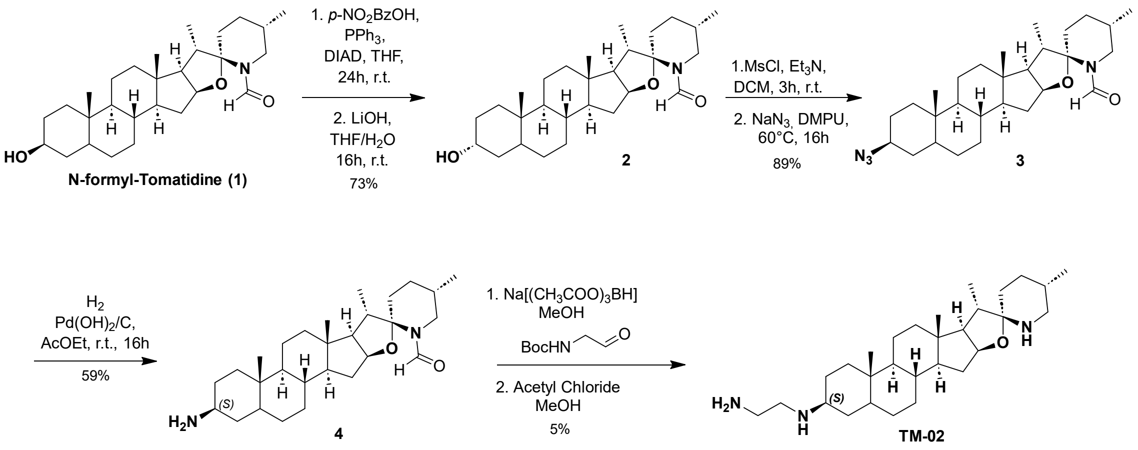

N-formyl Tomatidine **1** was synthesized following literature reported by Chagnon *et. al.*(http://dx.doi.org/10.1016/j.ejmech.2013.11.019)

Compound 2

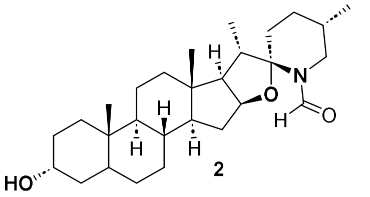

N-formyl Tomatidine **1** (1.50 g, 3.38 mmol) was dissolved in dry tetrahydrofuran (58 mL, 57 mM) under $Ar_{(g)}$ along with triphenylphosphine (1.77 g, 6.76 mmol) and 4-nitrobenzoic acid (1.58 g, 9.47 mmol). Diisopropyl-azodicarboxylate (1.35 mL, 6.76 mmol) was added and the reaction was stirred for 16h. The volatiles were removed under reduced pressure and the residue was suspended in water (50 mL) and extracted with ethyl acetate (3 x 70 mL). The combined organic phase was washed with brine (70 mL), dried over magnesium sulfate, and concentrated under reduced pressure. Purification by silica gel chromatography (20 to 60% AcOEt:Hex) afforded a white solid residue. The residue was dissolved in a 4:1 mixture of tetrahydrofuran: water (50 mL, 68 mM). Lithium hydroxide monohydrate (0.323 g, 13.5 mmol) was added at ambient temperature and the reaction was stirred for 16h. The reaction was diluted with ethyl acetate (120 mL) and the organic phase was washed with saturated ammonium chloride solution (80 mL), brine (80 mL), dried over magnesium sulfate and concentrated under reduced pressure. Purification by silica gel chromatography (30 to 75% AcOEt:Hex) yielded (*3R*)-N-formyl tomatidine **2** as a white solid (1.09 g, 73%).

^{1}H NMR (400 MHz, $CDCl_3$) δ (ppm) 8.41 (s, 1H), 4.32-4.24 (m, 1H), 4.17-4.09 (m, 1H), 4.06-4.00 (brs, 1H), 2.70-2.60 (m, 1H), 2.59-2.49 (m, 1H), 2.05-1.94 (m, 1H), 1.91-1.83 (m, 1H),

1.80-1.07 (m, 24H), 1.05 (d, 3H, J = 6.9 Hz), 0.90 (d, 3H, J = 5.8 Hz), 0.82 (s, 3H), 0.79 (s, 3H), 0.79-0.72 (m, 1H).

^{13}C NMR (101 MHz, $CDCl_3$) δ (ppm) 158.9 (**CH**), 97.8 (**C_q**), 78.6 (**CH**), 66.6 (**CH**), 62.5 (**CH**), 56.0 (**CH**), 54.4 (**CH**), 44.8 (**CH_2**), 41.4 (**C_q**), 40.4 (**CH_2**), 39.2 (**CH**), 37.0 (**CH**), 36.3 (**C_q**), 36.0 (**CH_2**), 35.1 (**CH**), 32.3 (**CH_2**), 32.2 (**CH_2**), 32.1 (**CH_2**), 30.9 (**CH**), 29.3 (**CH_2**), 29.1 (**CH_2**), 28.5 (**CH_2**), 27.7 (**CH_2**), 20.7 (**CH_2**), 19.1 (**CH_3**), 17.1 (**CH_3**), 15.4 (**CH_3**), 11.4 (**CH_3**).

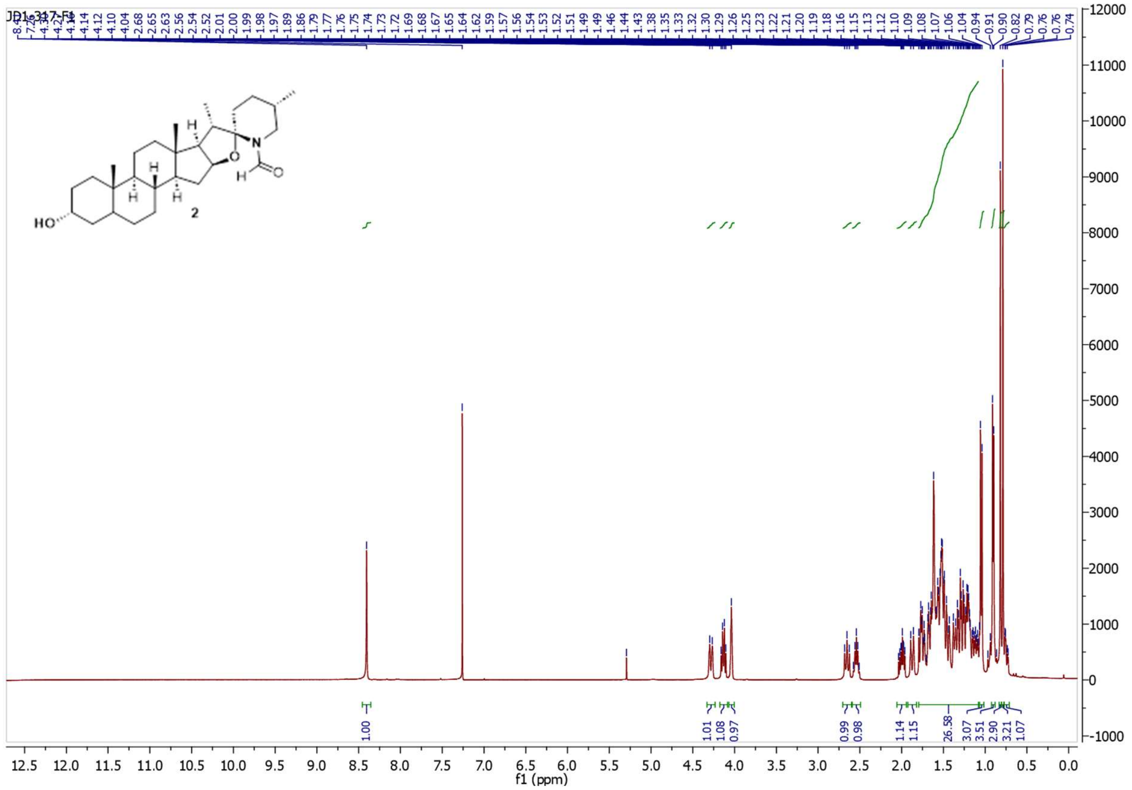

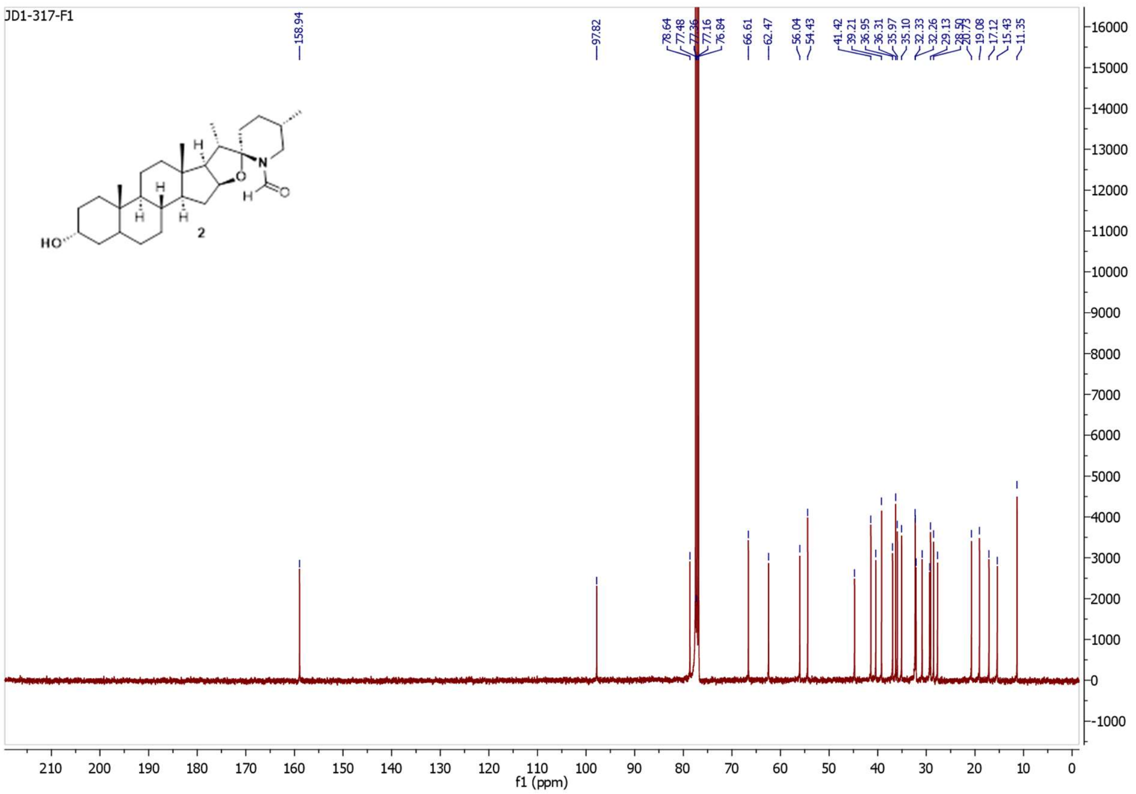

Compound 3

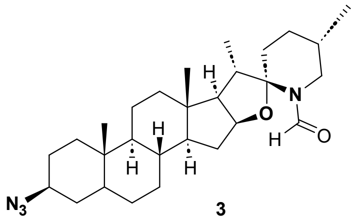

(*3R*)-N-formyl Tomatidine **2** (0.995 g, 2.24 mmol) was dissolved in dry dichloromethane (45 mL, 50 mM) under $Ar_{(g)}$. Triethylamine (0.469 mL, 3.36 mmol) and methanesulfonyl chloride (0.260 mL, 3.36 mmol) were added dropwise at ambient temperature and the mixture was stirred for 3h at ambient temperature. The reaction was quenched with saturated ammonium chloride solution (50 mL) and diluted with DCM (70 mL). The aqueous phase was extracted with DCM (3 x 50 mL) and the combined organic phase was washed with brine (80 mL), dried over magnesium sulfate, and concentrated under reduced pressure to afford a residue. The residue was dissolved in 1,3-dimethyl-3,4,5,6-tetrahydro-2-pyrimidinone (45 mL, 50 mM) under $Ar_{(g)}$. Sodium azide (0.729 g, 11.2 mmol) was added at ambient temperature and the reaction was heated at 60°C for 16h. After cooling to ambient temperature, water (80 mL) was added, and the white suspension was filtered. The solid was collected and filtrate was extracted with diethyl ether (2 x 110 mL). The organic phase was dried over magnesium sulfate and concentrated under reduced pressure. Purification of the residue by silica gel chromatography (5 to 70% AcOEt:Hex) yielded compound **3** as a white solid (0.936 g, 89%).

^{1}H NMR (400 MHz, $CDCl_3$) δ (ppm) 8.41 (s, 1H), 4.34-4.23 (m, 1H), 4.18-4.09 (m, 1H), 3.25 (tt, 1H, *J* = 4.6 Hz, *J* = 11.8 Hz), 2.69-2.61 (m, 1H), 2.58-2.49 (m, 1H), 2.03-1.94 (m, 1H), 1.90-1.07 (m, 22H), 1.05 (d, 3H, *J* = 6.9 Hz), 1.02-0.84 (m, 2H), 0.91 (d, 3H, *J* = 5.9 Hz), 0.82 (brs, 6H), 0.66 (td, 1H, *J* = 4.2 Hz, *J* = 12.1 Hz).

^{13}C NMR (101 MHz, $CDCl_3$) δ (ppm) 158.9 (**CH**), 97.8 (**C_q**), 78.6 (**CH**), 62.5 (**CH**), 60.6 (**CH**), 55.9 (**CH**), 54.3 (**CH**), 45.3 (**CH**), 44.7 (**CH_2**), 41.4 (**C_q**), 40.3 (**CH_2**), 37.2 (**CH_2**), 36.9 (**CH**), 35.7 (**C_q**), 35.0 (**CH**), 34.1 (**CH_2**), 32.2 (**CH_2**), 32.1 (**CH_2**), 30.8 (**CH**), 29.3 (**CH_2**), 28.5 (**CH_2**), 27.7 (**CH_2**), 21.1 (**CH_2**), 19.1 (**CH_3**), 17.1 (**CH_3**), 15.4 (**CH_3**), 12.4 (**CH_3**).

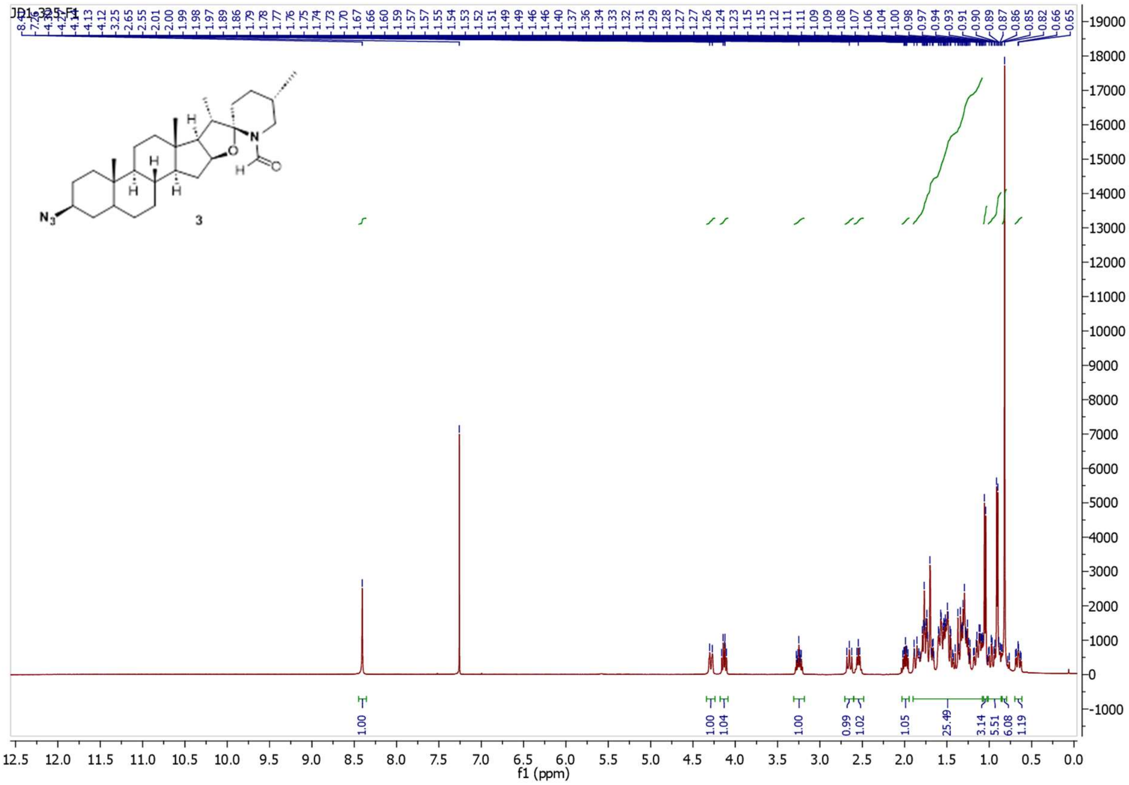

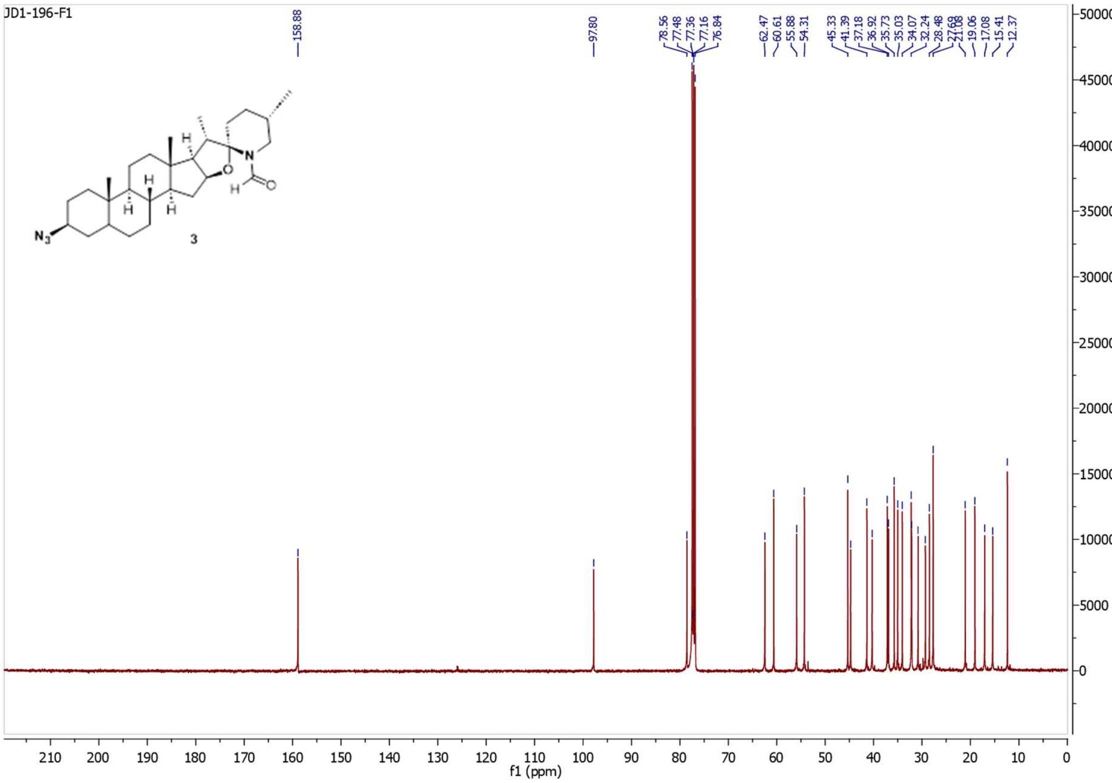

Compound 4

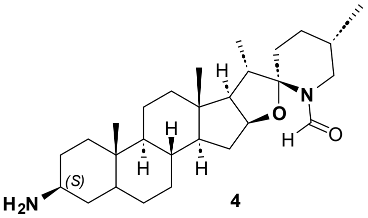

4

Compound **3** (0.910 g, 1.94 mmol) was dissolved in dry ethyl acetate (45.0 mL, 43 mM) under $Ar_{(g)}$. Palladium hydroxide, 20 wt. % Pd on carbon (0.136 g, 0.194 mmol) was added at ambient temperature. The mixture was degassed under sonication for 10 mins. Then, a balloon of $hydrogen_{(g)}$ was plugged, and the solution was stirred for 16h. at ambient temperature. Upon full conversion, the flask was flushed with $Ar_{(g)}$, the mixture diluted with ethyl acetate (80 mL) and the solution filtered on a pad of celite. The volatiles were removed under reduced pressure. Purification by silica gel chromatography (5 to 20% DCM:MeOH (10%NH_4OH)) yielded compound **4** as a white solid (0.509 g, 59%).

^{1}H NMR (400 MHz, CD_3OD) δ (ppm) 8.39 (s, 1H), 4.27-4.13 (m, 2H), 2.72-2.52 (m, 3H), 2.05-1.95 (m, 2H), 1.90-1.78 (m, 2H), 1.75-1.12 (m, 21H), 1.09 (d, 3H, *J* = 6.9 Hz), 1.06-0.94 (m, 2H), 0.92 (d, 3H, *J* = 5.9 Hz), 0.88 (s, 3H), 0.85 (s, 3H), 0.72 (td, 1H, *J* = 4.1 Hz, *J* = 12.2 Hz).

^{13}C NMR (101 MHz, CD_3OD) δ (ppm) 160.8 (**CH**), 99.2 (**C_q**), 79.9 (**CH**), 63.9 (**CH**), 57.0 (**CH**), 55.8 (**CH**), 51.9 (**CH**), 46.9 (**CH**), 45.9 (**CH_2**), 42.5 (**C_q**), 41.3 (**CH_2**), 39.1 (**CH_2**), 38.8 (**CH_2**),

38.2 (**CH**), 36.8 (**C_q**), 36.5 (**CH**), 33.5 (**CH_2**), 33.0 (**CH_2**), 32.3 (**CH_2**), 32.0 (**CH**), 30.2 (**CH_2**), 29.8 (**CH_2**), 28.7 (**CH_2**), 22.2 (**CH_2**), 19.4 (**CH_3**), 17.4 (**CH_3**), 15.7 (**CH_3**), 12.8 (**CH_3**).

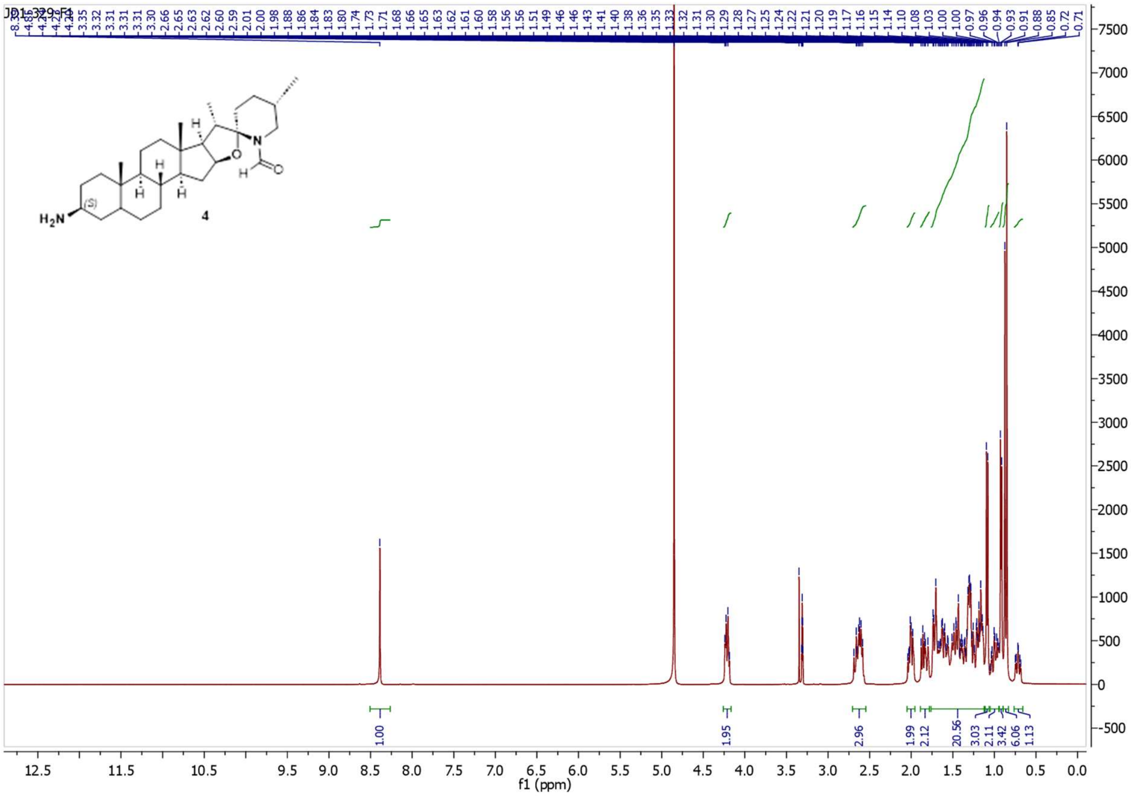

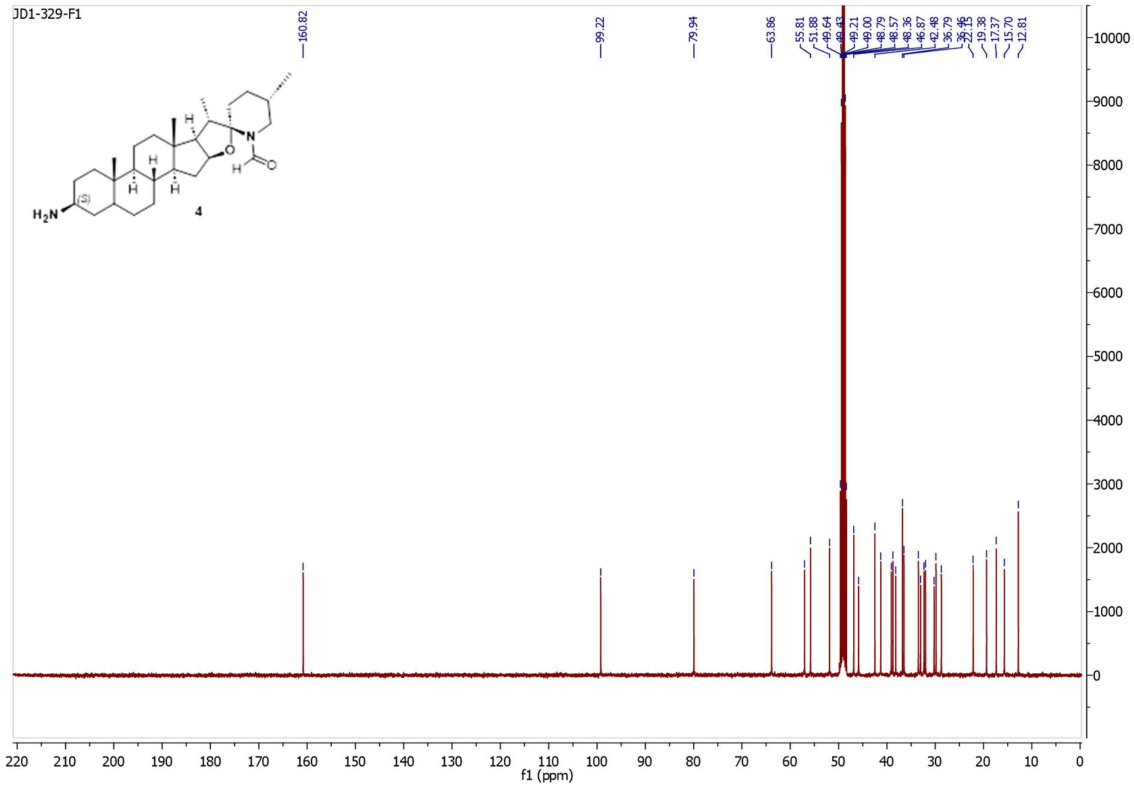

^{1}H NMR (400 MHz, $CDCl_3$) δ (ppm) 8.36 (s, 1H), 4.31-4.18 (m, 1H), 4.09 (dd, 1H, *J* = 7.1 Hz, *J* = 15.7 Hz), 2.72-2.56 (m, 2H), 2.55-2.38 (m, 2H), 1.99-1.90 (m, 1H), 1.87-1.78 (m, 1H), 1.77-1.00 (m, 24H), 1.01 (d, 3H, *J* = 6.9 Hz), 0.86 (d, 3H, *J* = 5.8 Hz), 0.78 (s, 3H), 0.76 (s, 3H), 0.61 (td, 1H, *J* = 3.9 Hz, *J* = 12.0 Hz).

^{13}C NMR (101 MHz, $CDCl_3$) δ (ppm) 158.8 (**CH**), 97.7 (**C_q**), 78.5 (**CH**), 62.4 (**CH**), 55.9 (**CH**), 54.4 (**CH**), 51.1 (**CH**), 45.5 (**CH**), 44.7 (**CH_2**), 41.3 (**C_q**), 40.3 (**CH_2**), 37.5 (**CH_2**), 36.8 (**CH**), 35.7 (**C_q**), 35.0 (**CH**), 32.3 (**CH_2**), 32.1 (**CH_2**), 30.8 (**CH**), 29.2 (**CH_2**), 28.6 (**CH_2**), 27.6 (**CH_2**), 21.0 (**CH_2**), 19.0 (**CH/CH_3**), 17.0 (**CH/CH_3**), 15.3 (**CH/CH_3**), 12.4 (**CH/CH_3**).

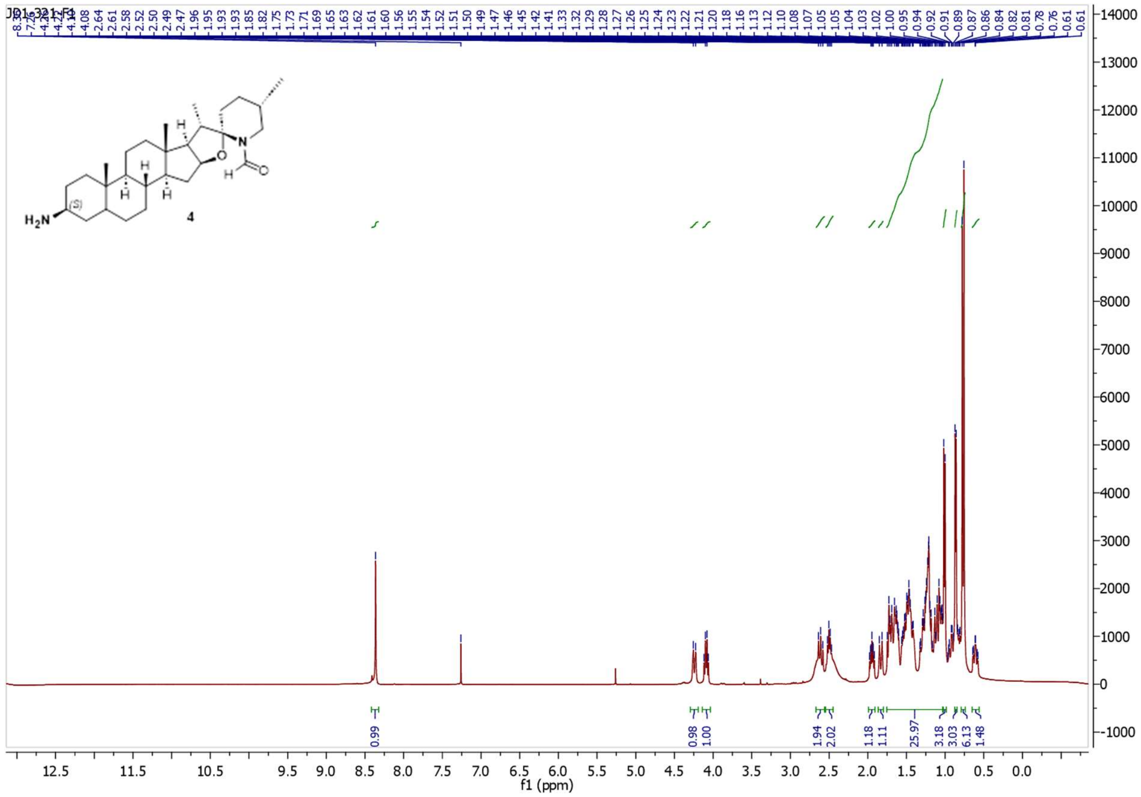

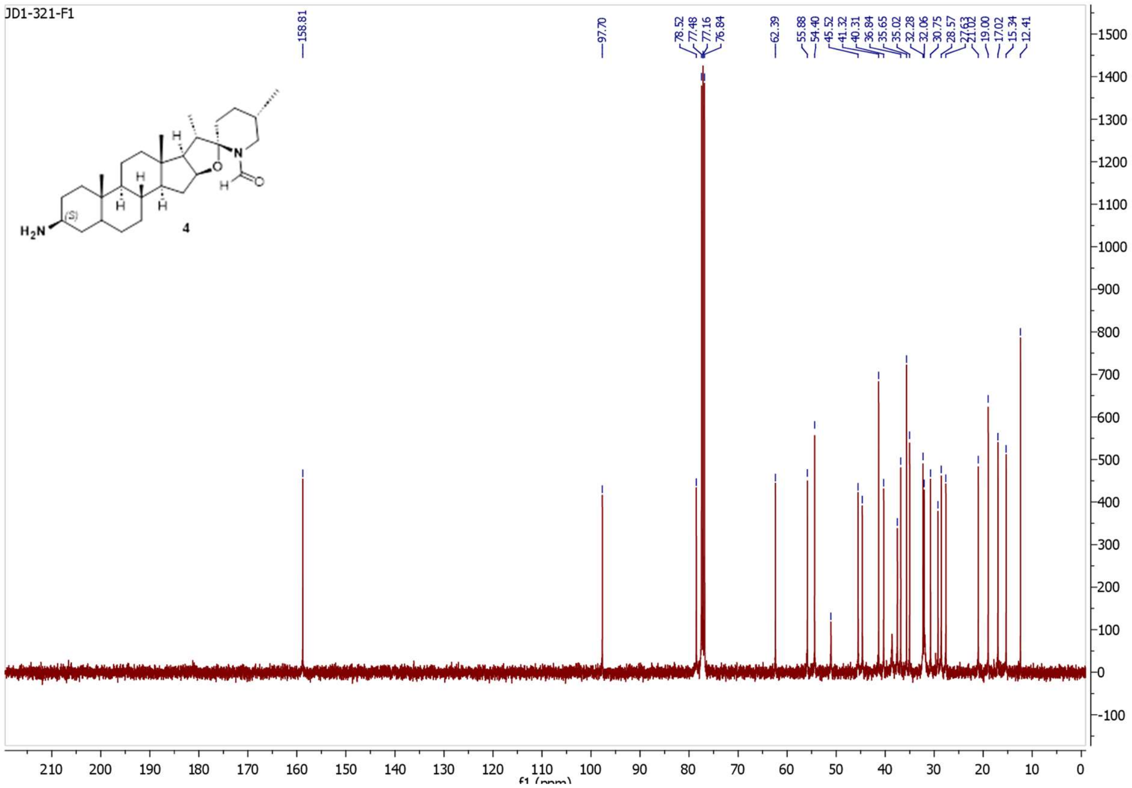

TM-02

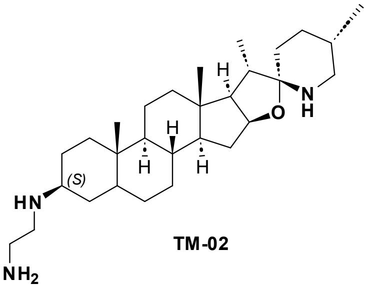

TM-02

Compound **4** (0.045g, 0.102 mmol) and N-boc-2-aminoacetaldehyde (0.032g, 0.203 mmol) were dissolved in methanol (6.7 mL, 15 mM) under $Ar_{(g)}$. The mixture was stirred for 5 mins at ambient temperature before addition of glacial acetic acid (one drop) and the reaction was stirred for another 1h at ambient temperature. Then, sodium triacetoxyborohydride (0.064g, 0.305 mmol) was added and the reaction was stirred for 1h at ambient temperature and at 65°C for 16h. The volatiles were removed under reduced pressure and the crude was suspended in saturated sodium bicarbonate solution (10 mL). The mixture was extracted with ethyl acetate (3 x 20 mL) and the combined organic phase was washed with brine, dried over magnesium sulfate and concentrated under reduced pressure. The residue was suspended in anhydrous methanol (3.0 mL). To the reaction mixture was added dropwise a solution of acetyl chloride (0.363 mL, 5.10 mmol) in anhydrous methanol (5.0 mL) previously stirred at ambient temperature for 0.5h. The reaction was heated at 65°C for 2h. and the volatiles were removed under reduce pressure. The residue was dissolved in a mixture of DMSO, CH_3CN and H_2O,

filtered and purified by MS preparative HPLC (0.5% formic acid) to yield compound **TM-02** as a colorless solid (0.022g, 5%).

^{1}H NMR (400 MHz, CD_3OD) δ (ppm) 8.31 (s, 0.1 H, **H**CO_2H), 4.39 (dd, 1H, *J* = 7.3 Hz, *J* = 15.6 Hz), 3.28-3.23 (m, 3H), 3.16-3.06 (m, 2H), 2.91 (t, 1H, *J* = 12.3 Hz), 2.25-2.17 (m, 1H), 2.09-1.94 (m, 4H), 1.92-1.13 (m, 26H), 1.11 (d, 3H, *J* = 7.0 Hz), 0.98 (d, 3H, *J* = 6.5 Hz), 0.93 (s, 3H), 0.90 (s, 3H), 0.77 (td, 1H, *J* = 3.8 Hz, *J* = 12.1 Hz).

^{13}C NMR (101 MHz, CD_3OD) δ (ppm) 97.6, 82.4, 63.1, 58.9, 56.8, 55.2, 49.3, 46.0, 43.1, 42.3, 42.2, 41.0, 37.6, 37.5, 36.8, 36.3, 33.2, 32.8, 32.5, 29.7, 29.5, 27.1, 27.0, 26.2, 22.1, 18.7, 17.3, 14.7, 12.5.

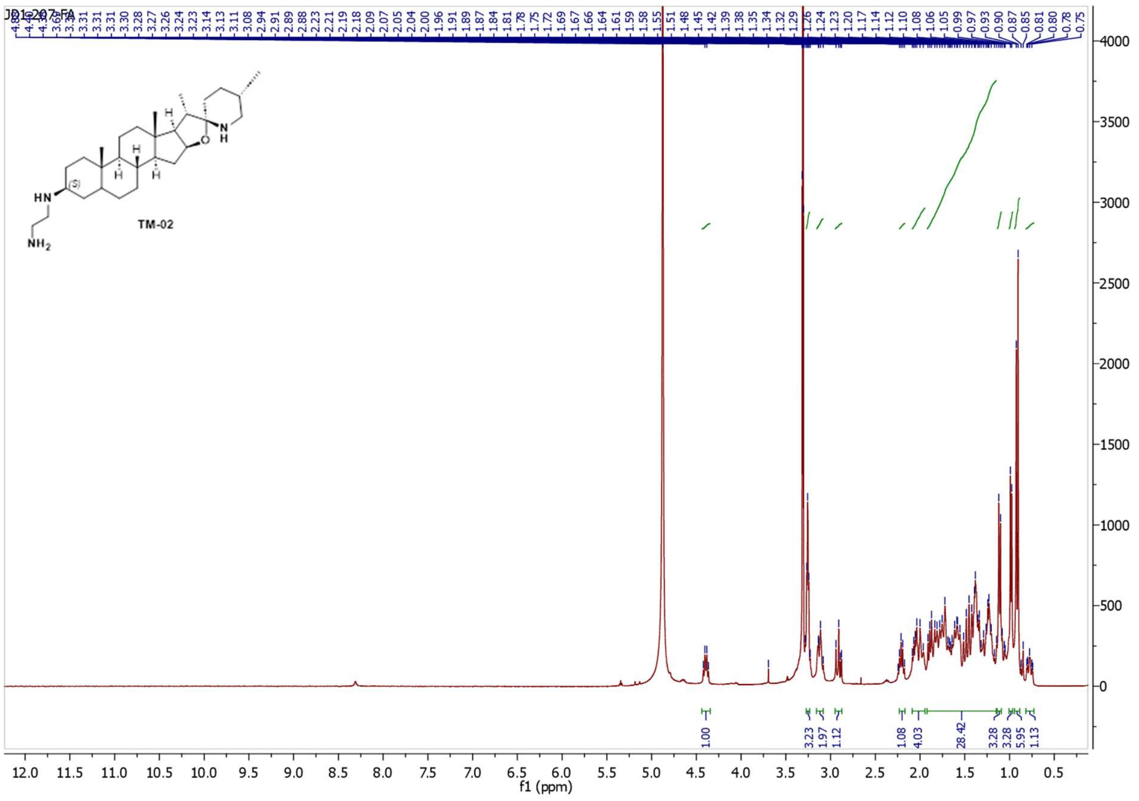

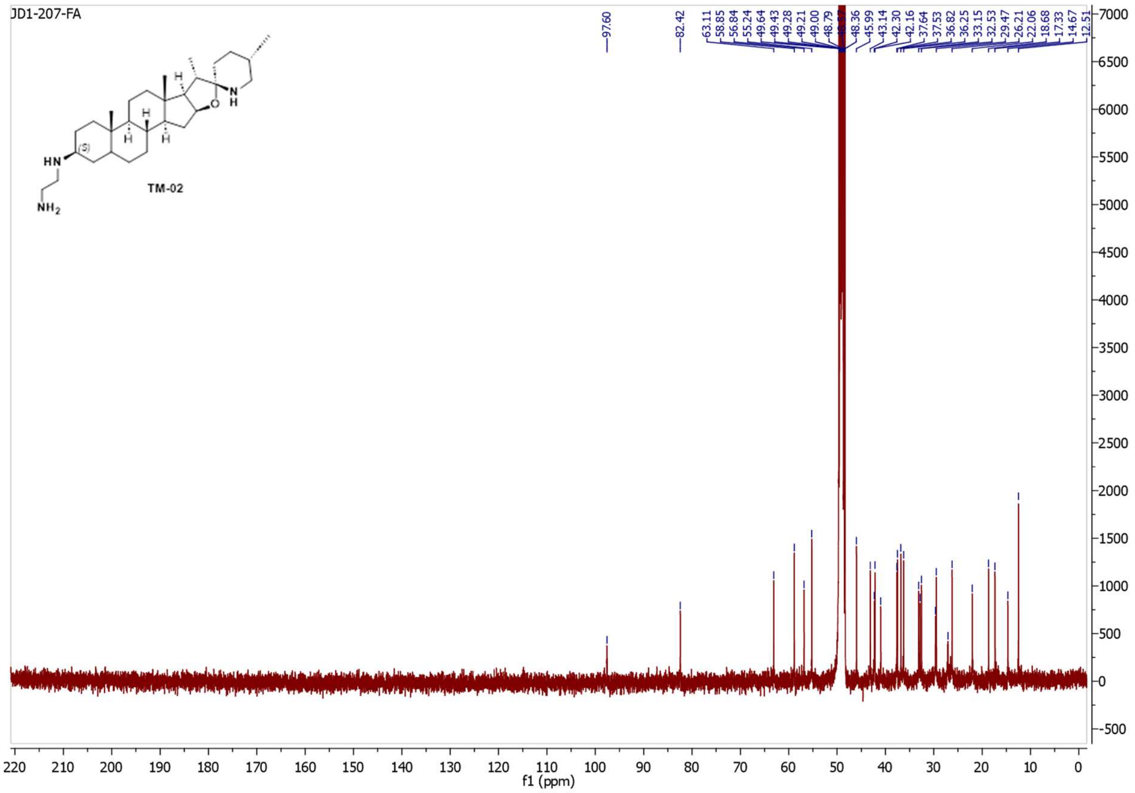

JD1-207-FA
TM-02
HN
NH2
N
H
O
(S)
97.60
82.42
63.11
58.85
56.84
55.24
49.64
49.43
49.28
49.21
49.00
48.79
48.36
45.99
43.14
42.30
42.16
37.64
37.53
36.82
36.25
33.15
32.53
29.47
26.21
22.06
18.68
17.33
14.67
12.51
f1 (ppm)
